# Supplementary material for: A general model-based causal inference method overcomes the curse of synchrony and indirect effect
Source: Nat Commun. 2023 Jul 24;14:4287. doi: 10.1038/s41467-023-39983-4 (PMC10366229; doi:10.1038/s41467-023-39983-4)
Supplement: Supplementary file 1 — Supplementary Information [file 41467_2023_39983_MOESM1_ESM.pdf]

A general model-based casual inference method overcomes the curse of synchrony and indirect effect

Se Ho Park[1,2], Seokmin Ha[2,3], and Jae Kyoung Kim[2,3,*]
[1]*Department of Mathematics, University of Wisconsin-Madison, WI 53706, United States*
[2]*Biomedical Mathematics Group, Institute for Basic Science, Daejeon 34126, Republic of Korea*
[3]*Department of Mathematical Sciences, KAIST, Daejeon 34141, Republic of Korea and*
[*]*jaekkim@kaist.ac.kr*

CONTENTS

I. Theoretical foundations 3
II. Regulation-detection function and score for 2D regulations 8
III. Framework for inferring a network with high dimensional regulations 9
IV. Description of the *in silico* models 10
V. Inference results from *in silico* data set 13
VI. Incorporating prior knowledge into the inference from GOBI 13
VII. Advantages of GOBI over model-based methods for predicting causal relationships 14
VIII. Optimizing the threshold values used in GOBI 15
IX. Distinguish indirect regulation using combined p-value 18
X. The robustness of GOBI to various types of noise 18
XI. Description of the experimental time-series data 20
XII. Inference results from experimental data set 21
XIII. The accuracy of GOBI on experimental data at different sampling rates 23
XIV. Extension to temporal-structured model including non-monotonic regulation 23
XV. Extension to infer time-delayed causal interactions 26
XVI. Guideline for determining whether the data is sufficient or not 27
XVII. Computational cost of GOBI 28
XVIII. Manual for the GOBI computational package 28

LIST OF FIGURES

1 Inferring 2D regulation types using regulation-detection functions and scores . . . 8
2 Framework for inferring a network with N number of components . . . 9
3 Inference of network structures under different assumptions of self-regulation types . . . 13
4 Comparing model-based method and GOBI for inferring a regulatory network . . . 14
5 Optimizing the threshold values used in GOBI . . . 16
6 Combined p-value can distinguish between direct and indirect regulations in the presence of noise . . . 18
7 The robustness of GOBI to various types of noise . . . 19
8 Approximate noise level of experimental data using residual . . . 21
9 Applying GOBI on experimental data sets to infer regulation . . . 22
10 Accuracy of GOBI on experimental data at different sampling rates . . . 24
11 Extended framework of GOBI to distinguish non-monotonic regulation from the absence of regulation . 25
12 Inferring delayed regulation using an extended regulation-detection score . . . 26
13 The required amount of data increases as the dimension of regulation increases . . . 28
14 Sample input and output for the GOBI package . . . 29

I. THEORETICAL FOUNDATIONS

In the main text, we inferred a regulation by deriving the conditions for interactions satisfying the general monotonic ODE. Specifically, the regulation-detection function captures the monotone relationship between causes and target, and the regulation-detection score quantifies the positiveness of the regulation-detection function. Then, we remove false positive predictions using the regulation-delta function. Furthermore, in the presence of noise, we relax the criteria for the regulation-detection score and develop a Total Regulation Score to infer the regulation. Also, we relax the criteria for the regulation-delta function and develop a surrogate test to distinguish indirect regulations. Here, we describe the theoretical foundation of our method.

A. Regulation-detection region, function, and score

Definition 1 (Dimension)**.** *For any regulation, the dimension of the regulation is defined as the number of causes. For example, if a target Y is regulated by N different number of causes $X_1, X_2, \cdots, X_N$, then it is called N dimensional (ND) regulation.*

If there is a ND regulation from $\mathbf{X} = (X_1, X_2, \cdots, X_N)$ to Y, then the dynamic of Y is given as

$$\frac{dY}{dt} = f(\mathbf{X}) = f(X_1, X_2, \cdots, X_N). \tag{1}$$

For each $i \in \{1, 2, \cdots, N\}$, we assume that X_i either positively ($X_i \to Y$) or negatively ($X_i \dashv Y$) regulates Y. If X_i positively (negatively) regulates Y, f is monotonically increasing (monotonically decreasing) with respect to X_i. For example, if X_i positively regulates Y, then for all fixed $\tilde{x}_j \in \text{range}(X_j)$ where $j = 1, 2, \cdots, N$ except for i, $f_i(\tilde{X}_j) : x_i \in \text{range}(X_i) \mapsto f(\tilde{x}_1, \cdots, \tilde{x}_{i-1}, x_i, \tilde{x}_{i+1}, \cdots, \tilde{x}_N)$ is monotonically increasing. Conversely, if X_i negatively regulates Y, $f_i(\tilde{X}_j)$ is monotonically decreasing.

Definition 2 (Regulation type)**.** *Suppose that there is an ND regulation from $\boldsymbol{X} = (X_1, X_2, \cdots, X_N)$ to Y. Then, its regulation type is denoted as an N-tuple $\sigma \in \{\pm\}^N$ where for each $i \in \{1, 2, \cdots, N\}$, $\sigma(i) = +$ ($\sigma(i) = -$) represents the positive (negative) regulation from X_i to Y.*

Remark 1. In the presence of self-regulation, X_N is Y in Eq. (1), and the dimension of regulation is the number of causes except Y.

Remark 2. There are 2^N different types of ND regulation.

From now on, we will assume that there is an ND regulation from $\mathbf{X} = (X_1, X_2, \cdots, X_N)$ to Y. We further assume that there are smooth time series of $X_1(t), X_2(t), \cdots, X_N(t), Y(t)$ on the domain $[0, \tau)$.

Definition 3. *For any $t, t^* \in [0, \tau)$, the difference of the time series between t and t^* is defined as*

$$\begin{aligned} X_i^d(t, t^*) &:= X_i(t) - X_i(t^*) \;\; \text{for each } i \in \{1, 2, \cdots, N\} \\ \dot{Y}^d(t, t^*) &:= \dot{Y}(t) - \dot{Y}(t^*). \end{aligned}$$

Using the difference in the time series, we can investigate the relationship between causes and target. For example, if there is a positive 1D regulation from X to Y, then the derivative of target Y (i.e., $\dot{Y}$) increases as the cause X increases. In other words, $\dot{Y}^d(t, t^*) > 0$ for any t, t^* satisfying $X^d(t, t^*) > 0$. The positive relationship can be quantified using the function $I_{X+}^Y = X^d \cdot \dot{Y}^d$. Specifically, in the presence of $X \to Y$, I_{X+}^Y is always positive on the region of t, t^* which satisfies $X^d(t, t^*) > 0$. This region and function can be extended for ND regulations as follows.

Definition 4 (Regulation-detection region)**.** *For the regulation type σ, regulation-detection region $R_{\boldsymbol{X}^\sigma} \subset [0, \tau)^2$ is defined as*

$$R_{\boldsymbol{X}^\sigma} := \{(t, t^*) \in [0, \tau)^2 \mid \sigma(i) X_i^d(t, t^*) > 0 \text{ for all } i \in \{1, 2, \cdots, N\}\}.$$

Definition 5 (Size of regulation-detection region)**.** *The size of regulation-detection region $R_{\boldsymbol{X}^\sigma}$ is defined as*

$$\text{size}(R_{\boldsymbol{X}^\sigma}) = \frac{\iint_{R_{\boldsymbol{X}^\sigma}} \mathbb{1} \, dt dt^*}{\iint_{[0,\tau)^2} \mathbb{1} \, dt dt^*}.$$

Remark 3. For ND regulation type σ from $\mathbf{X} = (X_1, X_2, \cdots, X_N)$ to Y, $\{R_{\mathbf{X}^\sigma}\}$ is a partition of $[0,\tau)^2$. By Remark 2, the number of partitions is 2^N, including the empty region, so the average of size$(R_{\mathbf{X}^\sigma})$ decreases exponentially as the dimension increases.

Definition 6 (Regulation-detection function)**.** *For any $(t,t^*) \in R_{\boldsymbol{X}^\sigma}$, regulation-detection function $I^Y_{\boldsymbol{X}^\sigma}$ is defined as*

$$I^Y_{\mathbf{X}^\sigma}(t,t^*) := \dot{Y}^d(t,t^*) \cdot \prod_{i=1}^{N} \sigma(i) X_i^d(t,t^*).$$

Then, the positive sign of regulation-detection function $I^Y_{\mathbf{X}^\sigma}$ is quantified by its normalized integral, regulation-detection score.

Definition 7 (Regulation-detection Score)**.** *The regulation-detection score of regulation type σ is defined as*

$$S^Y_{\mathbf{X}^\sigma} = \frac{\iint_{R_{\boldsymbol{X}^\sigma}} I^Y_{\boldsymbol{X}^\sigma}(t,t^*)dtdt^*}{\iint_{R_{\boldsymbol{X}^\sigma}} |I^Y_{\boldsymbol{X}^\sigma}(t,t^*)|dtdt^*}.$$

Theorem 1. *In the presence of ND regulation type σ from $\boldsymbol{X} = (X_1, X_2, \cdots, X_N)$ to Y, if $R_{\boldsymbol{X}^\sigma} \neq \varnothing$, then $S^Y_{\boldsymbol{X}^\sigma} = 1$.*

Proof. For fixed $(t,t^*) \in R_{\mathbf{X}^\sigma}$,

$$\begin{aligned}
\dot{Y}^d(t,t^*) &= \dot{Y}(t) - \dot{Y}(t^*) \\
&= f(X_1(t), X_2(t), \cdots, X_N(t)) - f(X_1(t^*), X_2(t^*), \cdots, X_N(t^*)) \\
&= f(X_1(t), X_2(t), X_3(t) \cdots, X_{N-1}(t), X_N(t)) - f(X_1(t^*), X_2(t), X_3(t) \cdots, X_{N-1}(t), X_N(t)) \\
&\quad + f(X_1(t^*), X_2(t), X_3(t), \cdots, X_{N-1}(t), X_N(t)) - f(X_1(t^*), X_2(t^*), X_3(t) \cdots, X_{N-1}(t), X_N(t)) \\
&\quad + \cdots \\
&\quad + f(X_1(t^*), X_2(t^*), X_3(t^*), \cdots, X_{N-1}(t^*), X_N(t)) - f(X_1(t^*), X_2(t^*), X_3(t^*) \cdots, X_{N-1}(t^*), X_N(t^*)) \\
&= \sum_{i=1}^{N} \left(f|_{X_i}(X_i(t)) - f|_{X_i}(X_i(t^*)) \right)
\end{aligned}$$

where

$$f|_{X_i}(p) = f(X_1(t^*), \cdots, X_{i-1}(t^*), p, X_{i+1}(t), \cdots, X_N(t))$$

for any $i \in \{1, 2, \cdots, N\}$. By the definition of the regulation type, $f|_{X_i}$ is a monotonic increasing (decreasing) function when $\sigma(i) = +$ ($\sigma(i) = -$). Therefore, if $\sigma(i) = +$ (i.e., X_i positively regulates Y), then

$$f|_{X_i}(X_i(t)) - f|_{X_i}(X_i(t^*)) > 0 \quad \text{for any} \quad (t,t^*) \in [0,\tau)^2 \text{ s.t. } X_i^d(t,t^*) > 0.$$

Conversely, if $\sigma(i) = -$ (i.e., X_i negatively regulates Y), then

$$f|_{X_i}(X_i(t)) - f|_{X_i}(X_i(t^*)) > 0 \quad \text{for any} \quad (t,t^*) \in [0,\tau)^2 \text{ s.t. } -X_i^d(t,t^*) > 0.$$

To sum up, by the definition of the regulation-detection region, if $R_{\mathbf{X}^\sigma}$ is not empty, then

$$f|_{X_i}(X_i(t)) - f|_{X_i}(X_i(t^*)) > 0 \quad \text{for any} \quad (t,t^*) \in R_{\mathbf{X}^\sigma}$$

for all $i \in \{1, 2, \cdots, N\}$. By using these positive relationships between causes and target on $R_{\mathbf{X}^\sigma}$,

$$\begin{aligned}
I^Y_{\mathbf{X}^\sigma}(t,t^*) &= \dot{Y}^d(t,t^*) \cdot \prod_{i=1}^{N} \sigma(i) X_i^d(t,t^*) \\
&= \left(\sum_{i=1}^{N} f|_{X_i}(X_i(t)) - f|_{X_i}(X_i(t^*)) \right) \cdot \prod_{i=1}^{N} \sigma(i) X_i^d(t,t^*) > 0.
\end{aligned}$$

Therefore, by the definition of the regulation-detection score,

$$S^Y_{\mathbf{X}^\sigma} = \frac{\iint_{R_{\mathbf{X}^\sigma}} I^Y_{\mathbf{X}^\sigma}(t,t^*)dtdt^*}{\iint_{R_{\mathbf{X}^\sigma}} |I^Y_{\mathbf{X}^\sigma}(t,t^*)|dtdt^*} = \frac{\iint_{R_{\mathbf{X}^\sigma}} I^Y_{\mathbf{X}^\sigma}(t,t^*)dtdt^*}{\iint_{R_{\mathbf{X}^\sigma}} I^Y_{\mathbf{X}^\sigma}(t,t^*)dtdt^*} = 1.$$

We conclude that if there is regulation type σ, then $R_{\mathbf{X}^\sigma} = \varnothing$ or $S^Y_{\mathbf{X}^\sigma} = 1$. $\square$

By Theorem 1, we can infer the regulation type σ using the criteria $S^Y_{\mathbf{X}^\sigma} = 1$ unless $R_{\mathbf{X}^\sigma} \neq \varnothing$.

In most biological systems, degradation of molecules increases as their own concentration increases. Therefore, in such cases, we assume that self-regulation is negative for every component in the system. In other words, X_N in Eq. (1) is Y and thus f is monotonically decreasing with respect to Y. With negative self-regulation, the regulation-detection region and function can be defined similarly.

B. Regulation-delta function

Although the regulation-detection score reflects the presence of regulation, it sometimes infers false positive predictions as in the following corollary.

Corollary 1. *Suppose that ND regulation type σ from $\boldsymbol{X} = (X_1, X_2, \cdots, X_N)$ to Y is present. Let us consider a new component X_{new} which does not regulate Y. Then, $S^Y_{\boldsymbol{X}^\sigma X^+_{\text{new}}} = 1$ unless $R_{\boldsymbol{X}^\sigma X^+_{\text{new}}} \neq \varnothing$ and $S^Y_{\boldsymbol{X}^\sigma X^-_{\text{new}}} = 1$ unless $R_{\boldsymbol{X}^\sigma X^-_{\text{new}}} \neq \varnothing$.*

Proof. Assume that $R_{\mathbf{X}^\sigma X^+_{\text{new}}} \neq \varnothing$ and fix $(t, t^*) \in R_{\mathbf{X}^\sigma X^+_{\text{new}}}$. Since $R_{\mathbf{X}^\sigma X^+_{\text{new}}} \subset R_{\mathbf{X}^\sigma}$, $(t, t^*) \in R_{\mathbf{X}^\sigma}$. By the definition of the regulation-detection function,

$$
\begin{aligned}
I^Y_{\mathbf{X}^\sigma X^+_{\text{new}}}(t, t^*) &= \dot{Y}^d(t, t^*) \cdot \left(\prod_{i=1}^{N} \sigma(i) X^d_i(t, t^*) \right) \cdot (+X^d_{\text{new}}(t, t^*)) \\
&= I^Y_{\mathbf{X}^\sigma}(t, t^*) \cdot X^d_{\text{new}}(t, t^*).
\end{aligned}
$$

Since $(t, t^*) \in R_{\mathbf{X}^\sigma}$, $I_{\mathbf{X}^\sigma}(t, t^*)$ is positive by the proof of Theorem 1. Also, $X^d_{\text{new}}(t, t^*)$ is positive since $(t, t^*) \in R_{\mathbf{X}^\sigma X^+_{\text{new}}}$. Thus, $I^Y_{\mathbf{X}^\sigma X^+_{\text{new}}}(t, t^*)$ is positive. Since this is true for all $(t, t^*) \in R_{\mathbf{X}^\sigma X^+_{\text{new}}}$, $S^Y_{\mathbf{X}^\sigma X^+_{\text{new}}} = 1$. Similarly, $S^Y_{\mathbf{X}^\sigma X^-_{\text{new}}} = 1$ unless $R_{\mathbf{X}^\sigma X^-_{\text{new}}} \neq \varnothing$. □

According to Corollary 1, in the presence of any subset of regulation type σ from $\mathbf{X}$ to Y, $S^Y_{\mathbf{X}^\sigma} = 1$ unless $R_{\mathbf{X}^\sigma} \neq \varnothing$. Thus, false positive regulations can be inferred by the criteria $S^Y_{\mathbf{X}^\sigma} = 1$. To prevent inferring those false positive regulations, we define the regulation-delta function which quantifies the effect of the new component on existing regulation. Specifically, in Corollary 1, $S^Y_{\mathbf{X}^\sigma X^+_{\text{new}}} = S^Y_{\mathbf{X}^\sigma X^-_{\text{new}}} = 1$ reflects that a new component (X_{new}) does not affect an existing regulation type σ. Thus, the difference between $S^Y_{\mathbf{X}^\sigma X^+_{\text{new}}}$ and $S^Y_{\mathbf{X}^\sigma X^-_{\text{new}}}$ can quantify the effect of the new component on existing regulation.

Definition 8 (Regulation-delta function)**.** *For an existing regulation from $\boldsymbol{X}$ to Y with regulation type σ and a new component X_{new}, regulation-delta function $\Delta^Y_{\boldsymbol{X}^\sigma}(X_{\text{new}})$ is defined as*

$$
\Delta^Y_{\boldsymbol{X}^\sigma}(X_{\text{new}}) := S^Y_{\boldsymbol{X}^\sigma X^+_{\text{new}}} - S^Y_{\boldsymbol{X}^\sigma X^-_{\text{new}}}.
$$

Simply, Δ is defined as the regulation-detection score when the new component positively affects the target minus the regulation-detection score when the new component negatively affects the target. Thus, if $\Delta = 0$ for all data, it suggests that the new component has no impact on the existing regulation, indicating a false positive prediction.

Corollary 2. *Suppose that there exists a regulation from $\boldsymbol{X}$ to Y with regulation type σ and let X_{new} be a new component. Then, $\Delta^Y_{\boldsymbol{X}^\sigma}(X_{\text{new}}) = 0$. In addition, in the presence of regulation with type $(\sigma, +)$ ($(\sigma, -)$) from $(\boldsymbol{X}, X_{\text{new}})$ to Y, then $\Delta^Y_{\boldsymbol{X}^\sigma}(X_{\text{new}}) \geq 0$ ($\Delta^Y_{\boldsymbol{X}^\sigma}(X_{\text{new}}) \leq 0$).*

Proof. The first statement holds by Corollary 1, because $S^Y_{\mathbf{X}^\sigma X^+_{\text{new}}} = S^Y_{\mathbf{X}^\sigma X^-_{\text{new}}} = 1$ in the presence of regulation type σ from $\mathbf{X}$ to Y. The second statement holds by Theorem 1, because $S^Y_{\mathbf{X}^\sigma X^+_{\text{new}}} = 1$ ($S^Y_{\mathbf{X}^\sigma X^-_{\text{new}}} = 1$) whereas $S^Y_{\mathbf{X}^\sigma X^-_{\text{new}}} \leq 1$ ($S^Y_{\mathbf{X}^\sigma X^+_{\text{new}}} \leq 1$) in the presence of regulation type $(\sigma, +)$ ($(\sigma, -)$) from $(\mathbf{X}, X_{\text{new}})$ to Y. □

After inferring the regulation using the criteria $S^Y_{\mathbf{X}^\sigma} = 1$, we remove the false positive regulation using the criteria $\Delta \neq 0$ for ≥2D regulations. For example, let σ be an ND regulation type from $\mathbf{X} = (X_1, X_2, \cdots, X_N)$ to Y with $S^Y_{\mathbf{X}^\sigma} = 1$. Let $\hat{\sigma}_i$ be the regulation type deleting ith component from σ, i.e. $\hat{\sigma}_i$ is $(N-1)$D regulation from $\hat{\mathbf{X}}_i := (X_1, \cdots, X_{i-1}, X_{i+1}, \cdots, X_N)$ to Y. Then, for all i, we compute $\Delta^Y_{\hat{\mathbf{X}}_i^{\hat{\sigma}_i}}(X_i)$ for each time-series data. If $\Delta^Y_{\hat{\mathbf{X}}_i^{\hat{\sigma}_i}}(X_i) \neq 0$ for some data, this indicates the presence of regulation type σ. Conversely, if $\Delta^Y_{\hat{\mathbf{X}}_i^{\hat{\sigma}_i}}(X_i) = 0$ for all the

data, then σ is identified as a false positive prediction. For example, let us consider the 3D regulation $\substack{X_1\to\\X_2\dashv\\X_3\to}Y$, which has been inferred from the criteria of regulation-detection score, $S^Y_{X_1^+X_2^-X_3^+} = 1$. To determine whether it is a false-positive prediction or not, we need to perform three Δ tests, each focusing on a different causal variable. Specifically, we compute $\Delta^Y_{X_1^+X_2^-}(X_3) = S^Y_{X_1^+X_2^-X_3^+} - S^Y_{X_1^+X_2^-X_3^-}$, $\Delta^Y_{X_1^+X_3^+}(X_2) = S^Y_{X_1^+X_2^+X_3^+} - S^Y_{X_1^+X_2^-X_3^+}$, and $\Delta^Y_{X_2^-X_3^+}(X_1) = S^Y_{X_1^+X_2^-X_3^+} - S^Y_{X_1^-X_2^-X_3^+}$. If they are not zero for some data, then the regulation $\substack{X_1\to\\X_2\dashv\\X_3\to}Y$ pass the Δ tests. However, if $\Delta^Y_{X_1^+X_2^-}(X_3)$ is zero for all data, $X_3 \to Y$ is a false-positive because X_3 does not affect an existing regulation $\substack{X_1\to\\X_2\dashv}Y$. Similarly, if $\Delta^Y_{X_1^+X_3^+}(X_2)$ and $\Delta^Y_{X_2^-X_3^+}(X_1)$ are zero, then $X_2 \dashv Y$ and $X_1 \to Y$ are false-positive predictions, respectively.

C. Inferring regulation from noisy time series

In the presence of noise, we cannot directly apply Theorem 1 (Fig. 3b). Thus, we relax the criteria of the regulation-detection region and the score when noise is present. We first relax the criteria of the regulation-detection region from $R_{\mathbf{X}^\sigma} \neq \varnothing$ to size$(R_{\mathbf{X}^\sigma}) > R^{\text{thres}}$. In other words, we consider $R_{\mathbf{X}^\sigma}$ as an empty set when its size is smaller than the certain threshold R^{thres}. Next, the criteria of the regulation-detection score is relaxed from $S^Y_{\mathbf{X}^\sigma} = 1$ to $S^Y_{\mathbf{X}^\sigma} > S^{\text{thres}}$. We will discuss optimizing the thresholds later in Section VIII. By using these relaxed criteria, we provide a new measure for inferring the regulations from the noisy time series.

Definition 9 (Total regulation score)**.** *For a regulation type σ from $\boldsymbol{X}$ to Y, among the data which has non-empty regulation-detection region (i.e., $R_{\mathbf{X}^\sigma} > R^{\text{thres}}$), the fraction of satisfying the relaxed criteria $S^Y_{\mathbf{X}^\sigma} > S^{\text{thres}}$ is called Total Regulation Score (*$\text{TRS}^Y_{\mathbf{X}^\sigma}$*).*

Remark 4. TRS is the measure that integrates the advantage and penalty functions of the regulation-detection score with respect to the size of the regulation-detection region. Specifically, the criteria $S^Y_{\mathbf{X}^\sigma} > S^{\text{thres}}$ is more difficult to satisfy when $R_{\mathbf{X}^\sigma}$ is large. Thus, data that satisfy the criteria are scored higher when the region is large. Conversely, for data that do not satisfy the criteria, a higher penalty is given for the smaller region. Thus, we use both the advantage function (f) and the penalty function (g) to score the data. In addition, h is a function for filtering the empty region, i.e., $h(\text{size}(R_{\mathbf{X}^\sigma})) = 0$ when $R_{\mathbf{X}^\sigma} < R^{\text{thres}}$.

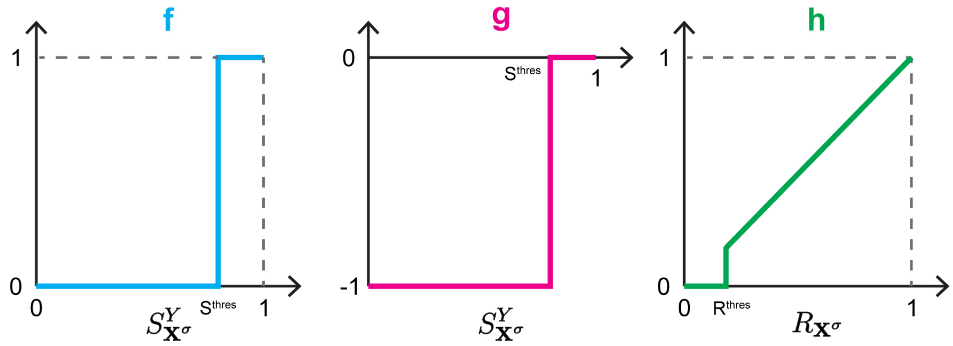

$f(S^Y_{\mathbf{X}^\sigma}) = 1$ when the regulation-detection score satisfies the criteria $S^Y_{\mathbf{X}^\sigma} > S^{\text{thres}}$, and $f(S^Y_{\mathbf{X}^\sigma}) = 0$ otherwise. Then, we can give an advantage proportional to the size of the regulation-detection region as follows.

$$f(S^Y_{\mathbf{X}^\sigma}) \cdot h(\text{size}(R_{\mathbf{X}^\sigma})).$$

Also, $g(S^Y_{\mathbf{X}^\sigma}) = -1$ when the regulation-detection score does not satisfy the criteria $S^Y_{\mathbf{X}^\sigma} > S^{\text{thres}}$, and $g(S^Y_{\mathbf{X}^\sigma}) = 0$ otherwise. Then, we can give a penalty inversely proportional to the size of the regulation-detection region as

$$g(S^Y_{\mathbf{X}^\sigma}) \cdot (\alpha - h(\text{size}(R_{\mathbf{X}^\sigma}))$$

for some α which is larger than the maximum value of size$(R_{\mathbf{X}^\sigma})$. Now, we develop a measure (M) that combines

advantage and penalty. Assume that there are L number of data.

$$
\begin{aligned}
M &= \frac{\sum_{i=1}^{L} f(S^Y_{\mathbf{X}^\sigma}) \cdot h(\text{size}(R_{\mathbf{X}^\sigma}))}{\sum_{i=1}^{L} h(\text{size}(R_{\mathbf{X}^\sigma}))} + k \cdot \frac{\sum_{i=1}^{L} g(S^Y_{\mathbf{X}^\sigma}) \cdot (\alpha - h(\text{size}(R_{\mathbf{X}^\sigma})))}{\sum_{i=1}^{L} (\alpha - h(\text{size}(R_{\mathbf{X}^\sigma})))} \\
&= \frac{\sum_{S^Y_{\mathbf{X}^\sigma} > S^{\text{thres}}} h(\text{size}(R_{\mathbf{X}^\sigma}))}{\sum_{i=1}^{L} h(\text{size}(R_{\mathbf{X}^\sigma}))} + k \cdot \frac{\sum_{S^Y_{\mathbf{X}^\sigma} \le S^{\text{thres}}} -(\alpha - h(\text{size}(R_{\mathbf{X}^\sigma})))}{\sum_{i=1}^{L} (\alpha - h(\text{size}(R_{\mathbf{X}^\sigma})))}.
\end{aligned}
$$

Here, k is the weight of the penalty compared to the advantage. Let $A = \sum_{i=1}^{L} h(\text{size}(R_{\mathbf{X}^\sigma}))$ and choose $\alpha = \frac{k+1}{L} A$. We can choose the proper k such that α is larger than the maximum size of the regulation-detection region $R_{\mathbf{X}^\sigma}$. Then,

$$
\begin{aligned}
M &= \frac{\sum_{S^Y_{\mathbf{X}^\sigma} > S^{\text{thres}}} h(\text{size}(R_{\mathbf{X}^\sigma}))}{A} + \frac{\sum_{S^Y_{\mathbf{X}^\sigma} \le S^{\text{thres}}} h(\text{size}(R_{\mathbf{X}^\sigma}))}{A} - \frac{\sum_{S_{\mathbf{X}^\sigma} \le S^{\text{thres}}} \alpha}{A} \\
&= 1 - \frac{k+1}{L} \cdot (\text{the number of data satisfying } S^Y_{\mathbf{X}^\sigma} \le S^{\text{thres}}) \\
&\propto \frac{\text{the number of data satisfying } S^Y_{\mathbf{X}^\sigma} > S^{\text{thres}}}{L} = \text{TRS}^Y_{\mathbf{X}^\sigma}.
\end{aligned}
$$

Thus, TRS is the measure that integrates the advantage and penalty functions of the regulation-detection score with respect to the size of the regulation-detection region.

In summary, in the presence of noise, we use the criteria $\text{TRS}^Y_{\mathbf{X}^\sigma} > \text{TRS}^{\text{thres}}$ to infer the regulation type σ from $\mathbf{X}$ to Y. After that, false positive regulations are identified via Δ test. Specifically, from all the data, if the criteria $\Delta^Y_{\hat{\mathbf{X}}_i^{\hat{\sigma}_i}}(X_i) \ge 0$ when $\sigma(i) = +$ ($\Delta^Y_{\hat{\mathbf{X}}_i^{\hat{\sigma}_i}}(X_i) \le 0$ when $\sigma(i) = -$) is satisfied for all i, then the regulation type σ passes the Δ test. When the number of data is large enough ($\ge$25), the overall sign of Δ is measured using a one-tailed Wilcoxon signed rank test. Finally, we next perform the surrogate test, which is described in the main text, to remove indirect regulations.

II. REGULATION-DETECTION FUNCTION AND SCORE FOR 2D REGULATIONS

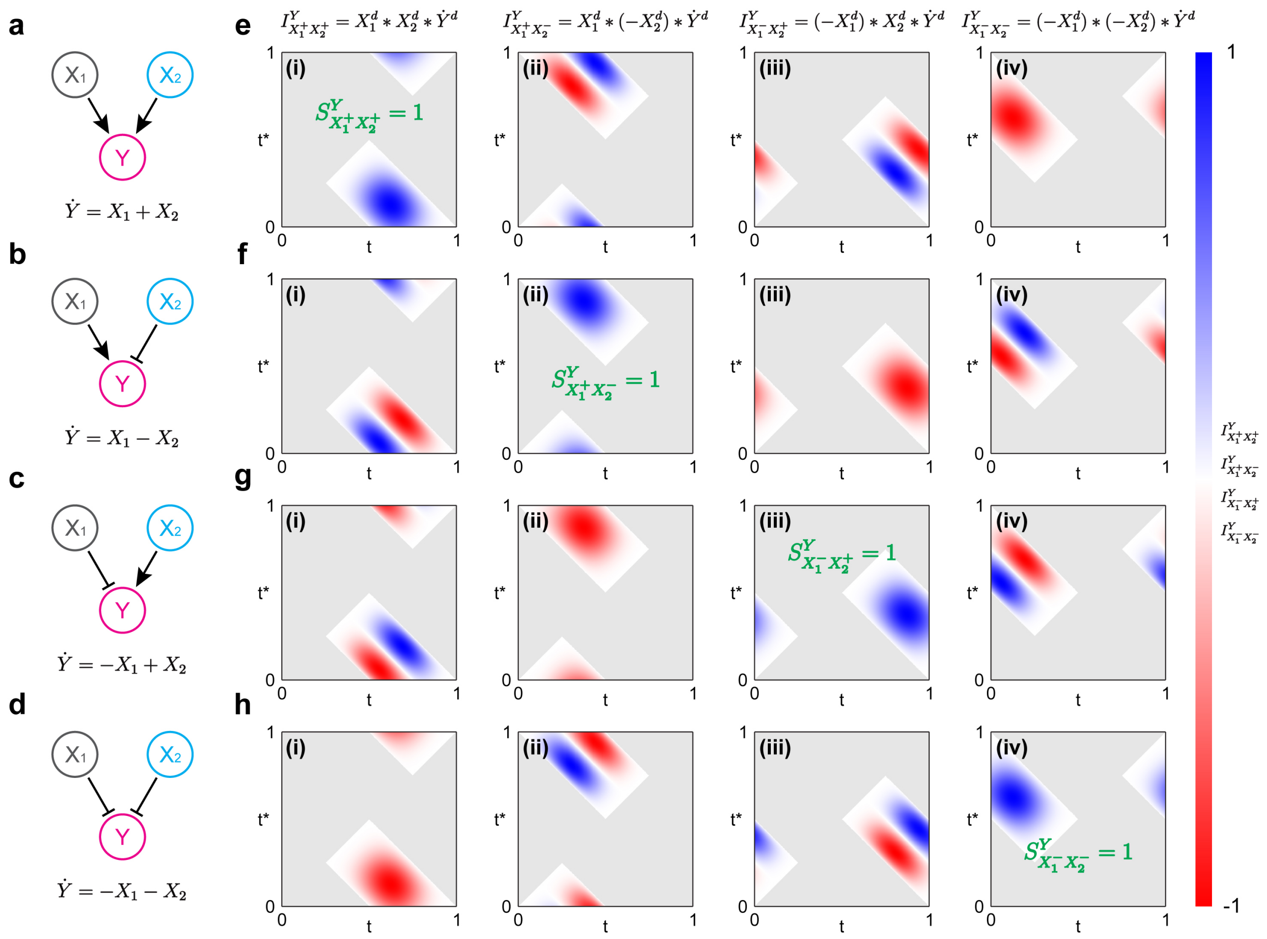

Supplementary Fig. 1. Inferring 2D regulation types using regulation-detection functions and scores. **a-d** Four types of 2D regulations from X_1 and X_2 to Y: ${}^{X_1 \to}_{X_2 \to} Y$ (**a**); ${}^{X_1 \to}_{X_2 \dashv} Y$ (**b**); ${}^{X_1 \dashv}_{X_2 \to} Y$ (**c**); and ${}^{X_1 \dashv}_{X_2 \dashv} Y$ (**d**). In terms of regulation type (σ), each 2D regulation can be written as $\sigma = (+,+)$ (**a**), $\sigma = (+,-)$ (**b**), $\sigma = (-,+)$ (**c**), and $\sigma = (-,-)$ (**d**). **e-h** In the presence of regulation type σ, the regulation-detection function, i.e., $I^Y_{X_1^{\sigma(1)} X_2^{\sigma(2)}} := (\sigma(1)X_1^d) \cdot (\sigma(2)X_2^d) \cdot \dot{Y}^d$ is always positive on $R_{\mathbf{X}^\sigma}$. For example, in the presence of regulation ${}^{X_1 \to}_{X_2 \dashv} Y$ (**b**), as X_1 increases and X_2 decreases, $\dot{Y}$ increases. Thus, $I^Y_{X_1^+ X_2^-} = X_1^d \cdot (-X_2^d) \cdot \dot{Y}^d$ is always positive when $X_1^d > 0$ and $X_2^d < 0$ (**f** (ii)). Such positivity disappears for the regulation-detection functions, which do not match with the actual regulation type (i.e., $I^Y_{X_1^+ X_2^+}$, $I^Y_{X_1^- X_2^+}$, and $I^Y_{X_1^- X_2^-}$ are not always positive) (**f** (i), (iii), and (iv)). Source data are provided as a Source Data file.

In the main text, two types of 2D regulations were described (Fig. 1d-i). Here, we describe the regulation-detection function and score of all the types of 2D regulations from X_1 and X_2 to Y (Supplementary Fig. 1a-d). For each type of 2D regulation type σ, we first define the regulation-detection region $R_{\mathbf{X}^\sigma}$ (Def. 4) (Supplementary Fig. 1e-h non-grey colored region). Next, we compute the regulation-detection function $I^Y_{\mathbf{X}^\sigma}$ (Def. 6) on $R_{\mathbf{X}^\sigma}$ (Supplementary Fig. 1e-h). In the presence of regulation type σ, the regulation-detection function, i.e., $I^Y_{X_1^{\sigma(1)} X_2^{\sigma(2)}} := (\sigma(1)X_1^d) \cdot (\sigma(2)X_2^d) \cdot \dot{Y}^d$ is always positive on $R_{\mathbf{X}^\sigma}$ (Supplementary Fig. 1e (i), f (ii), g (iii), and h (iv)). Note that such positivity disappears for the regulation-detection functions which do not match with the actual regulation type (Supplementary Fig. 1e (ii, iii, and iv), f (i, iii, and iv), g (i, ii, and iv), and h (i, ii, and iii)). The positivity and negativity of the regulation-detection function reflect the presence and absence of regulation, respectively. The sign of the function can be quantified with its normalized integral, regulation-detection score $S^Y_{\mathbf{X}^\sigma}$ (Def. 7). Thus, $S^Y_{\mathbf{X}^\sigma} = 1$ in the presence of regulation type σ (Thm. 1) (Supplementary Fig. 1e (i), f (ii), g (iii), and h (iv)).

III. FRAMEWORK FOR INFERRING A NETWORK WITH HIGH DIMENSIONAL REGULATIONS

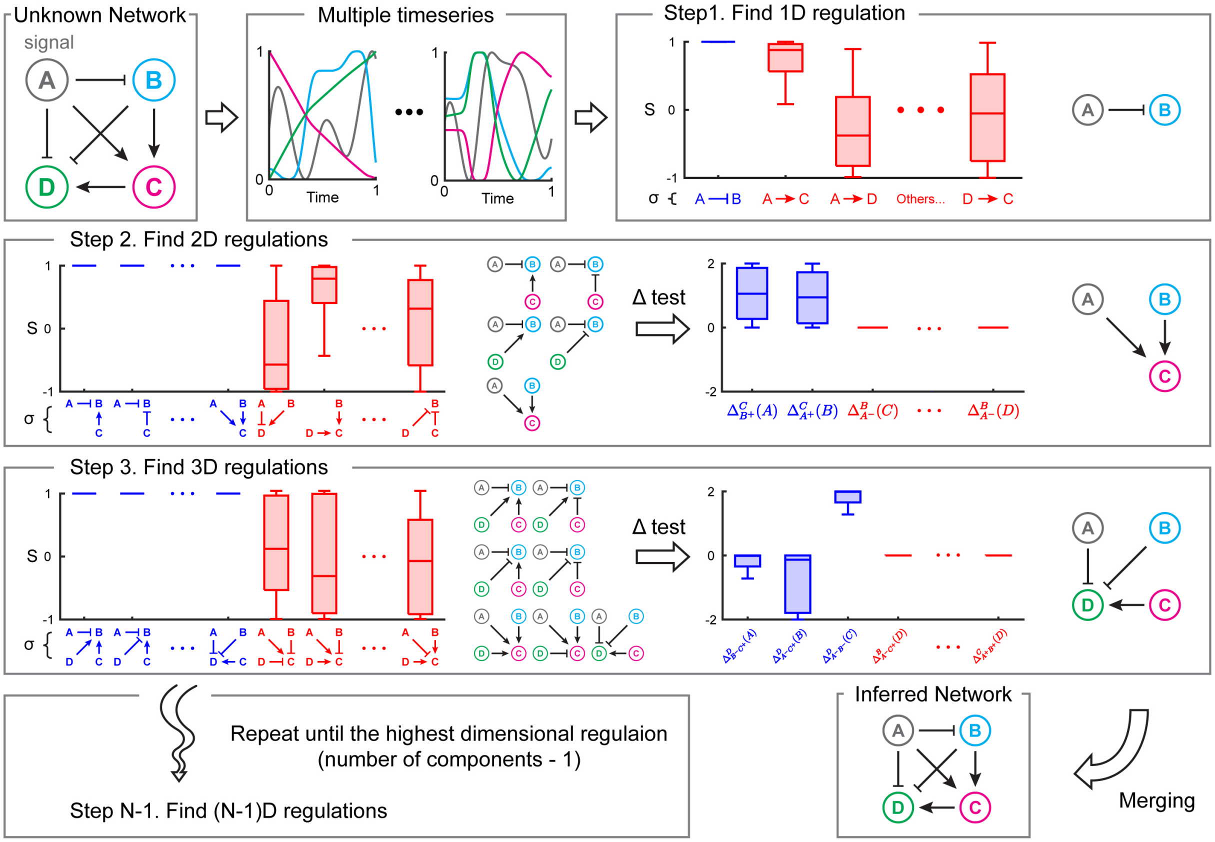

Supplementary Fig. 2. Framework for inferring a network with N number of components. With ODE describing the network (upper-left), various time series are simulated with different initial conditions (upper-middle). Then, from each time series, regulation-detection score $S^{Y}_{X^{\sigma}}$ is calculated for every 1D regulation type σ (Step 1). The criteria $S^{Y}_{X^{\sigma}} = 1$ infers $A \dashv B$. Next, $S^{Y}_{\mathbf{X}^{\sigma}}$ is calculated for every 2D regulation type σ. Among the five types of 2D regulations with $S^{Y}_{\mathbf{X}^{\sigma}} = 1$, only one passes the Δ test (Step 2). Lastly, $S^{Y}_{\mathbf{X}^{\sigma}}$ is calculated for every 3D regulation type σ. Among the seven types of 3D regulations with $S^{Y}_{\mathbf{X}^{\sigma}} = 1$, only one passes the Δ test (Step 3). Here, data are presented as box plots (n = 100), in which the box bounds the IQR divided by the median, and whiskers extend to a maximum of 1.5 × IQR beyond the box. If there are N components in the system, we go up to $(N-1)$D regulations (Step $N-1$), which is the highest possible dimension (lower-left). Because there are four components in this example, we go up to 3D regulation. By merging inferred 1∼3D regulations from Step 1∼3, the regulatory network is successfully inferred (bottom right). Source data are provided as a Source Data file.

$S^{Y}_{\mathbf{X}^{\sigma}} = 1$ (Def. 7) together with $\Delta^{Y}_{\hat{\mathbf{X}}^{\hat{\sigma}_i}}(X_i) \neq 0$ (Def. 8) can be used as an indicator of regulation type σ. Based on this, we construct an extended framework for inferring a regulatory network from time-series data when there are N components in the system (Supplementary Fig. 2). To illustrate this, we first obtain random signal A. Specifically, we randomly choose nine different numbers from $[-1, 1]$ to determine the value of $A(t)$ when $t = 0, \frac{1}{8}, \cdots, \frac{7}{8}, 1$ and connect the nine values using the MATLAB function 'spline'. Then, we simulate the following ODE on $[0, 1)$ with initial conditions of B, C, and D randomly selected from $[-1, 1]$.

$$\frac{dB}{dt} = -A^3$$
$$\frac{dC}{dt} = A^5 + B^3$$
$$\frac{dD}{dt} = -0.5A^5 - 1.5B^3 - B^5 + C^3.$$

From each time series, regulation-detection score $S^{Y}_{X^{\sigma}}$ is calculated for every 1D regulation type σ from X to Y

($X, Y = A, B$, or C) (Step 1). Because only $A \dashv B$ satisfies the criteria $S^{Y}_{X^\sigma} = 1$ for every time series, only $A \dashv B$ is inferred as 1D regulation (Step 1). Next, $S^{Y}_{\mathbf{X}^\sigma}$ is calculated for every 2D regulation type σ (Step 2, left). Five types of regulations ($\substack{A\dashv\\C\to}B$, $\substack{A\dashv\\C\dashv}B$, $\substack{A\dashv\\D\to}B$, $\substack{A\dashv\\D\dashv}B$, and $\substack{A\to\\B\to}C$) satisfy the criteria $S^{Y}_{\mathbf{X}^\sigma} = 1$ for every time series. Among these, we can identify the false positive regulations by using the regulation-delta function (Step 2, right). $\Delta^{B}_{A^-}(C)$ is equal to zero for every time series, indicating that $\substack{A\dashv\\C\to}B$ and $\substack{A\dashv\\C\dashv}B$ are false positive regulations. Also, $\Delta^{B}_{A^-}(D)$ is equal to zero for every time series, indicating that $\substack{A\dashv\\D\to}B$ and $\substack{A\dashv\\D\dashv}B$ are false positive regulations. Thus, $\substack{A\to\\B\to}C$ is the only inferred 2D regulation as it satisfies the criteria for the regulation-delta function ($\Delta^{C}_{A^+}(B) \neq 0$ and $\Delta^{C}_{B^+}(A) \neq 0$). Lastly, $S^{Y}_{\mathbf{X}^\sigma}$ is calculated for every 3D regulation type σ (Step 3, left). Seven types of regulations satisfy the criteria $S^{Y}_{\mathbf{X}^\sigma} = 1$ for every time series. Note that most of them include the inferred 1D and 2D regulations ($A \dashv B$ and $\substack{A\to\\B\to}C$). Among these, we can identify the false positive regulations by using a regulation-delta function (Step 3, right). $\substack{A\dashv\\B\dashv\\C\to}D$ is the only inferred 3D regulation as it satisfies $\Delta^{D}_{A^-B^-}(C) \neq 0$, $\Delta^{D}_{A^-C^+}(B) \neq 0$, and $\Delta^{D}_{B^-C^+}(A) \neq 0$. This iterative process, i.e., infer the regulations using the criteria $S^{Y}_{\mathbf{X}^\sigma} = 1$ and remove false positive predictions using the criteria $\Delta \neq 0$, is repeated until the maximum possible dimension. Specifically, if there are N number of components in the system, we go up to $(N-1)$D regulations (Step $N-1$). Because there are four components in this system, the maximum possible dimension is 3. Thus, we go up to 3D regulation. By merging all the inferred regulations, the regulatory network is successfully inferred.

IV. DESCRIPTION OF THE *IN SILICO* MODELS

Here, we describe the various *in silico* models used as examples for our method (Fig. 2, 3).

A. Kim-Forger Model

The Kim-Forger model consists of a negative feedback loop that models mammalian circadian clocks (Fig. 2b) [1]. The model describes protein product represses the transcription of its mRNA by a protein sequestration mechanism. The model is given by the following system of ODEs.

$$
\begin{aligned}
\frac{dM}{dt} &= k_1 f(P) - k_2 M \\
\frac{dP_C}{dt} &= k_1 M - k_3 P_C \\
\frac{dP}{dt} &= k_1 P_C - k_4 P \\
f(z) &= \frac{A_t - K_d - z + \sqrt{(A_t - K_d - P)^2 - 4A_t K_d}}{2A_t}.
\end{aligned}
$$

Here, the variable M is the repressor $mRNA$ concentration; P_C is the cytosolic repressor protein concentration; P is the nuclear repressor protein concentration. As in [1], we simulate the Kim-Forger model with the parameters $k_1 = 1$, $k_2 = 0.16$, $k_3 = 0.29$, $k_4 = 0.3$, $A_t = 0.6$, and $K_d = 10^{-5}$.

B. Frzilator

The *frzilator* model consists of a negative feedback loop that models the oscillatory behavior of the Frz signal transduction system in *Myxococcus xanthus* (Fig. 2c) [2]. The model is given by the following system of ODEs.

$$
\begin{aligned}
\frac{df}{dt} &= \phi\left(\frac{1-f}{0.01 + (1-f)}\right) - d_f\left(\frac{f}{0.005 + f}\right)e \\
\frac{dc}{dt} &= k_c\left(\frac{1-c}{0.005 + (1-c)}\right)f - d_c\left(\frac{c}{0.005 + c}\right) \\
\frac{de}{dt} &= k_e\left(\frac{1-e}{0.005 + (1-e)}\right)c - d_e\left(\frac{e}{0.005 + e}\right).
\end{aligned}
$$

Here, the variable f is the fraction of activated FrzF; c is the fraction of methylated FrzCD; e is the fraction of phosphorylated FrzE. As in [2], we simulate the *frzilator* model with the parameters $\phi = 0.08$, $k_c = 4$, $k_e = 4$, $d_f = 1$, $d_c = 2$, and $d_e = 3$.

C. 4-state Goodwin oscillator

The Goodwin model describes the action of a protein product repressing its mRNA (Fig. 2d) ([3]). The model is given by the following system of ODEs.

$$\begin{aligned}
\frac{dM}{dt} &= \frac{1}{1+P_3^n} - \alpha_M M \\
\frac{dP_1}{dt} &= M - \alpha_1 P_1 \\
\frac{dP_2}{dt} &= P_1 - \alpha_2 P_2 \\
\frac{dP_3}{dt} &= P_2 - \alpha_3 P_3.
\end{aligned}$$

We simulate the Goodwin model with the parameters $\alpha_1 = 0.4$, $\alpha_2 = 0.4$, $\alpha_3 = 0.4$, $\alpha_M = 0.4$, and $n = 10$.

D. Goldbeter oscillator

The Goldbeter model describes circadian oscillations of the period protein (PER) in *Drosophila* (Fig. 2f) [4]. The model is based on multiple phosphorylations of PER and on the negative feedback exerted by nuclear PER on the transcription of the period gene. The model is given by the following system of ODEs.

$$\begin{aligned}
\frac{d[mRNA]}{dt} &= \frac{v_s}{1+([PER_n]/K_I)^n} - \frac{v_m[mRNA]}{K_{m1}+[mRNA]} \\
\frac{d[PER_0]}{dt} &= k_s[mRNA] - \frac{V_1[PER_0]}{K_1+[PER_0]} + \frac{V_2[PER_1]}{K_2+[PER_1]} \\
\frac{d[PER_1]}{dt} &= \frac{V_1[PER_0]}{K_1+[PER_0]} - \frac{V_2[PER_1]}{K_2+[PER_1]} - \frac{V_3[PER_1]}{K_3+[PER_1]} + \frac{V_4[PER_2]}{K_4+[PER_2]} \\
\frac{d[PER_2]}{dt} &= \frac{V_3[PER_1]}{K_3+[PER_1]} - \frac{V_4[PER_2]}{K_4+[PER_2]} - k_1[PER_2] + k_2[PER_N] - \frac{v_d[PER_2]}{K_d+[PER_2]} \\
\frac{d[PER_n]}{dt} &= k_1[PER_2] - k_2[PER_N].
\end{aligned}$$

Here, the variable $mRNA$ is the mRNA concentration of period gene; PER_0 is unphosphorylated PER; PER_1 is phosphorylated PER; PER_2 is fully phosphorylated PER; and PER_n is nuclear PER. As in [4], we simulate the Goldbeter model with the parameters $v_s = 0.76\mu M/h, v_m = 0.65\mu M/h, v_d = 0.95\mu M/h, k_s = 0.38h^{-1}, k_1 = 1.9h^{-1}, k_2 = 1.3h^{-1}, V_1 = 3.2\mu M/h, V_2 = 1.58\mu M/h, V_3 = 5\mu M/h, V_4 = 2.5\mu M/h, K_1 = K_2 = K_3 = K_4 = 1\mu M, K_I = 1\mu M, K_{m1} = 0.5\mu M, K_d = 0.2\mu M$, and $n = 4$.

E. cAMP oscillator

In *Dictyostelium*, pulses of adenosine 3′,5′-monophosphate (cAMP) are generated during specific stages of development. The cAMP oscillator model describes underlying signal transduction between cAMP and various protein

kinases (Fig. 2g) [5]. The model is given by the following system of ODEs.

$$\begin{aligned}
\frac{d[ACA]}{dt} &= k_1[CAR1] - k_2[ACA][PKA] \\
\frac{d[PKA]}{dt} &= k_3[cAMPi] - k_4[PKA] \\
\frac{d[ERK2]}{dt} &= k_5[CAR1] - k_6[PKA][ERK2] \\
\frac{d[RegA]}{dt} &= k_7 - k_8[ERK2][RegA] \\
\frac{d[cAMPi]}{dt} &= k_9[ACA] - k_{10}[RegA][cAMPi] \\
\frac{d[cAMPe]}{dt} &= k_{11}[ACA] - k_{12}[cAMPe] \\
\frac{d[CAR1]}{dt} &= k_13[cAMPe] - k_{14}[CAR1].
\end{aligned}$$

Here, the variable ACA is the adenylyl cyclase; PKA is the cAMP-dependent protein kinase A; $ERK2$ is the MAP kinase; $RegA$ is the intracellular cAMP phosphodiesterase; $cAMPi$ is the intracellular cAMP; $cAMPe$ is the extracellular cAMP; and $CAR1$ is the G protein–coupled cAMP receptor. As in [5], we simulate the cAMP oscillator model with the parameters $k_1 = 2.0$, $k_2 = 0.9$, $k_3 = 2.5$, $k_4 = 1.5$, $k_5 = 0.6$, $k_6 = 0.8$, $k_7 = 1.0$, $k_8 = 1.3$, $k_9 = 0.3$, $k_{10} = 0.8$, $k_{11} = 0.7$, $k_{12} = 4.9$, $k_{13} = 23$, and $k_{14} = 4.5$.

F. Feed-forward Loop (FFL)

Feed-foward loop (FFL) is one of the network motifs which recur throughout the transcription networks [6]. FFL, a three-gene pattern, is composed of two input transcription factors, one of which regulates the other, both jointly regulating a target gene. Here, three types of FFL, coherent FFL (CFL), incoherent FFL (IFL), and single FFL (SFL) are used. FFL is coherent (incoherent) when the sign of the direct regulation path is the same (opposite) as the sign of the indirect regulation path, respectively. SFL is when there is only one direct regulation path. Here, we describe the FFLs when A and B are the input transcription factors with A regulating B, and C is the target transcription gene. We use the following ODE system for CFL,

$$\begin{aligned}
\frac{dB}{dt} &= -A \\
\frac{dC}{dt} &= B - 0.2A - A^3
\end{aligned}$$

IFL,

$$\begin{aligned}
\frac{dB}{dt} &= -A^3 \\
\frac{dC}{dt} &= B + 2A^3
\end{aligned}$$

and SFL

$$\begin{aligned}
\frac{dB}{dt} &= -A \\
\frac{dC}{dt} &= 0.5 + B.
\end{aligned}$$

V. INFERENCE RESULTS FROM *IN SILICO* DATA SET

The results of our framework on *in silico* data set are described in Supplementary Data 1. For each system, we compute the regulation-detection score for every possible set of causes and target variables. Then, among the data, the ratio satisfying the criteria $S^{Y}_{\mathbf{X}^{\sigma}} = 1$ can be obtained and is listed in Supplementary Data 1. When the ratio is equal to one, this indicates that all the data satisfy the criteria, thus inferred as a direct regulation. The green box in the list highlights the inferred regulations (i.e., ratio = 1). For ≥2D regulations, among inferred regulations, we use the criteria of regulation-delta function ($\Delta \neq 0$) to detect the false positive predictions. The red box in the lists in Supplementary Data 1 indicates that the ratio = 1 but $\Delta = 0$, which means false positive regulations. As a result, we successfully infer all the true regulations from various systems using our framework. Here, we used 100 time-series data to compute 1D and 2D regulation-detection scores and 500 data to compute the 3D regulation-detection score.

VI. INCORPORATING PRIOR KNOWLEDGE INTO THE INFERENCE FROM GOBI

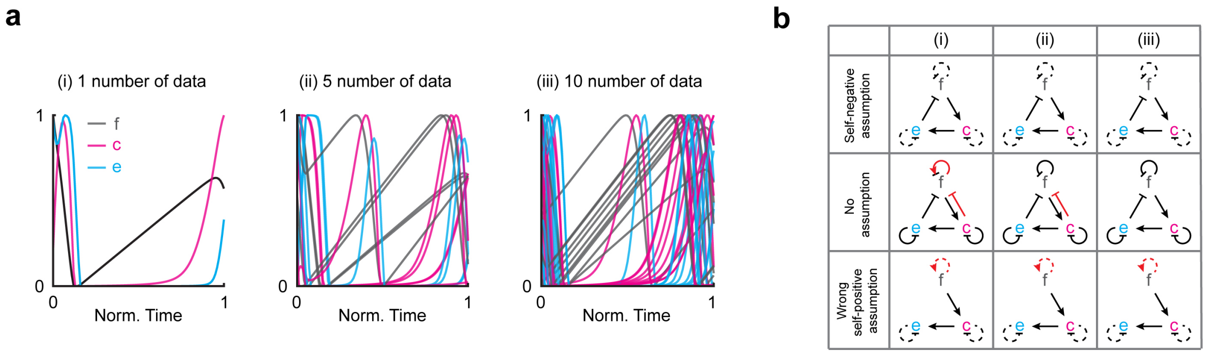

Supplementary Fig. 3. Inference of network structures under different assumptions of self-regulation types. **a** The Frzilator ODE model (Fig. 2c) is used to simulate one (**a** (i)), five (**a** (ii)), and ten (**a** (iii)) time series from different initial conditions. **b** Using these time-series data, the inference results from GOBI with three different approaches are illustrated: assuming negative self-regulations (**b** the first row), not assuming the types of self-regulation (**b** the second row), and assuming the wrong types of self-regulation (**b** the third row), where dashed lines indicate the assumed self-regulation types. In the last approach, the positive self-regulation of f is assumed, which is incorrect. Assuming negative self-regulation allows GOBI to accurately infer the network structure regardless of the amount of data (**b** the first row). However, not assuming the types of self-regulation leads to false positive predictions (red arrows) when the amount of data is limited (**b** the second row). Under wrong assumptions of self-regulation types, GOBI only infers regulations targeting components whose self-regulations are correctly assumed (**b** the third row). Source data are provided as a Source Data file.

When using GOBI for inference, users have the option to incorporate prior knowledge into the analysis. This involves assuming the types of regulation for specific parts of the network based on any available prior knowledge they have, which allows effective inference with a limited amount of data. In particular, in biological systems, the degradation rates of molecules typically increase as their own concentrations increase, so users can often assume the negative self-regulation as described in our manuscript. Of course, wrong assumptions may result in some incorrect inference results. To illustrate this, we use the example of the Frzilator (Fig. 2c) to infer the network structure with and without assuming the types of self-regulation.

Using the Frzilator ODE model, we first simulate one, five, and ten time series from different initial conditions which lie in the range of the original limit cycle (Supplementary Fig. 3a (i)-(iii)). From the time-series data, GOBI successfully infers the true network structure regardless of the amount of data when we assume the negative self-regulation (Supplementary Fig. 3b, the first row). However, without assuming self-regulation (Supplementary Fig. 3b, the second row), the inference results differ depending on the amount of data. Specifically, when ten time-series data are used, GOBI successfully infers the network structure. However, as the amount of data used decreases, the inferred network has some incorrect predictions (Supplementary Fig. 3b, red arrows in the second row). Taken together, assuming the types of self-regulation is not necessary, but it can be a useful option when the amount of available data is limited.

However, making wrong assumptions about the types of self-regulation can lead to some incorrect inference results. To investigate this, we apply GOBI by falsely assuming a positive self-regulation of f and correctly assuming negative self-regulations of c and e (Supplementary Fig. 3b, the third row). While GOBI successfully infers $f \to c$ and $c \to e$,

GOBI fails to infer regulation $e \dashv f$. This indicates that GOBI can incorrectly infer the regulation targeting the component whose self-regulation is incorrectly assumed.

VII. ADVANTAGES OF GOBI OVER MODEL-BASED METHODS FOR PREDICTING CAUSAL RELATIONSHIPS

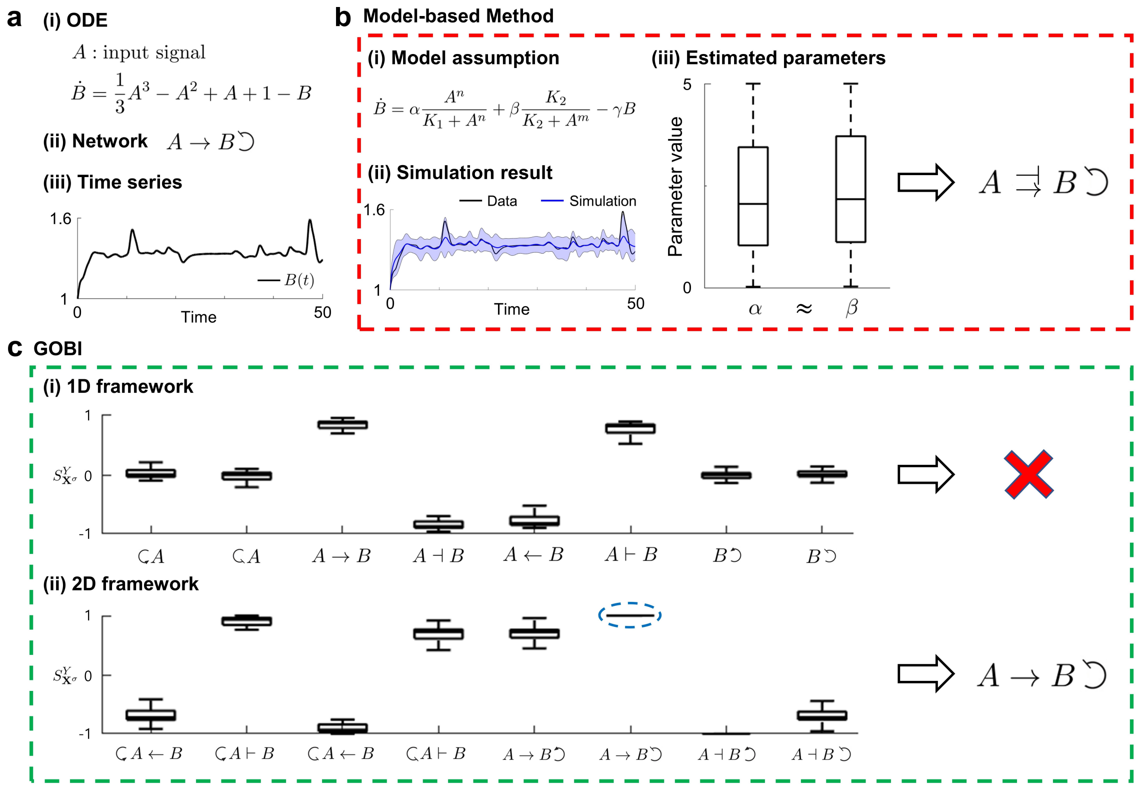

Supplementary Fig. 4. Comparing model-based method and GOBI for inferring a regulatory network. **a** The ODE (**a** (i)) describes a regulatory network (**a** (ii)) with two components. Here, A positively regulates B (i.e., $\frac{1}{3}A^3 - A^2 + A + 1$ is an increasing function with respect to A). With the ODE describing the network, the time series of B is simulated with initial condition $B(0) = 1$ with the time series of input signal A, which is constructed by connecting randomly selected 51 points from $\mathcal{N}(1, 0.5)$ over the time domain $[0, 50]$ using the spline fitting. (**a** (iii)). **b** Model-based method is tested with assuming two different hill functions ($\alpha\frac{A^n}{K_1+A^n}$ and $\beta\frac{K_2}{K_2+A^m}$) to describe the positive and negative regulation from A to B (**b** (i)). We use simulated annealing to estimate seven parameters $\alpha, \beta, \gamma, K_1, K_2, n$, and m. Here, parameters are selected within a range of $\alpha, \beta, \gamma \in [0, 5], K_1, K_2 \in [0, 20]$, and $n, m \in [1, 5]$. For 100 pairs of parameters that reproduce the time series data (**b** (ii)), the values of α and β are similar and positive (**b** (ii)) indicating that the regulation from A to B is a mixture. Thus, the model-based method fails to inter the network structure. Here, data are presented as box plots (n = 100), in which the box bounds the IQR divided by the median, and whiskers extend to a maximum of 1.5 × IQR beyond the box. **c** We then apply GOBI to infer the network structure. To apply GOBI, we first use the moving-window technique with a window size of 10 and an overlapping ratio of 10% to divide the time series into 41 different segments. Then, from each time-series data, $S^Y_{\mathbf{X}^\sigma}$ is computed for every 1D and 2D regulation (**c** (i)-(ii) left). The criteria $S^Y_{\mathbf{X}^\sigma} = 1$ infers no 1D regulation (**c** (i) right), and one 2D regulation (**c** (ii) right). This 2D regulation passes the Δ test and GOBI successfully infers the network structure. Here, data are presented as box plots (n = 41), in which the box bounds the IQR divided by the median, and whiskers extend to a maximum of 1.5 × IQR beyond the box. Source data are provided as a Source Data file.

In this section, we demonstrate how GOBI can be more effective than model-based approaches for predicting causal relationships. Model-based methods have a significant limitation in that their inference results depend on the choice of model. If an inappropriate mathematical model is chosen, it can lead to false predictions. For example, when attempting to model a positive regulation from A to B ($A \to B$), there are various ways to express this relationship mathematically. If an unsuitable model is chosen, the fitting of the model can inform the wrong causal relationship. However, GOBI does not require explicit knowledge of the underlying mathematical model and is thus free from such assumptions. To illustrate this advantage, we use a simple system with two components A and B (Supplementary

Fig. 4a (i)-(ii)). Here, A positively regulates B since $\frac{1}{3}A^3 - A^2 + A + 1$ is an increasing function with respect to A. With the ODE describing the network, we simulate a time-series data with initial condition of $B(0) = 1$ and input signal A (Supplementary Fig. 4a (iii)). The time series of input signal A is constructed by connecting 51 randomly selected points from $\mathcal{N}(1, 0.5)$ over the time domain [0, 50] with the spline fitting.

Suppose we do not know the underlying dynamics of this system and used the following hill-type functions to test for the existence of regulation from A to B (Supplementary Fig. 4b (i)), which has been widely used in the previous model-based inference [7–9]:

$$\dot{B} = \alpha \frac{A^n}{K_1 + A^n} + \beta \frac{K_2}{K_2 + A^m} - \gamma B.$$

This ODE includes both positive ($\alpha \frac{A^n}{K_1+A^n}$) and negative ($\beta \frac{K_2}{K_2+A^m}$) regulation from A to B. To estimate the parameters $\alpha, \beta, \gamma, K_1, K_2, n$, and m, we use simulated annealing with parameter ranges of $\alpha, \beta, \gamma \in [0, 5], K_1, K_2 \in [0, 20]$, and $n, m \in [1, 5]$. We collect 100 parameter sets that reproduce the time-series data (Supplementary Fig. 4b (ii)). The values of the parameters corresponding to positive regulation (α) and negative regulation (β) from A to B are similar, indicating the presence of both positive and negative regulation from A to B (Supplementary Fig. 4b (iii)). Consequently, the model-based method fails to detect the positive regulation from A to B. This example highlights the importance of selecting the correct underlying model to achieve accurate predictions of causal relationships using model-based methods, which can limit the effectiveness of these approaches.

We then apply GOBI, which does not need to specify underlying regulatory functions, to the same data. First, we employ the moving-window technique to divide the time series into 41 different segments with a length of 10, where each segment overlaps with the previous one by 10%. From each time-series data, we compute the regulation-detection scores for all possible 1D and 2D regulations (Supplementary Fig. 4c (i)-(ii) left). None of the 1D regulations pass the criteria $S^{Y}_{X+} = 1$ (Supplementary Fig. 4c (i) right), while one 2D regulation passes the criteria (Supplementary Fig. 4c (ii) right). Also, $\Delta^{B}_{A+}(B), \Delta^{B}_{B-}(A) \neq 0$ indicating that this 2D regulation is not a false positive regulation. By merging the results, GOBI successfully recovers the underlying regulatory network. This example illustrates the effectiveness of GOBI in cases where the underlying regulatory functions are unknown, in contrast to model-based methods that may fail under such circumstances.

VIII. OPTIMIZING THE THRESHOLD VALUES USED IN GOBI

In the presence of noise, we only consider the regulation-detection region which is not small (i.e., size($R_{\mathbf{X}^\sigma}$) $> R^{\text{thres}}$). On the regulation-detection region, we compute the regulation-detection score. In the presence of noise, the criteria for the regulation-detection score $S^{Y}_{\mathbf{X}^\sigma} = 1$ is relaxed to $S^{Y}_{\mathbf{X}^\sigma} > S^{\text{thres}}$. The fraction of data that satisfies the relaxed criteria ($S^{Y}_{\mathbf{X}^\sigma} > S^{\text{thres}}$) is $\text{TRS}^{Y}_{\mathbf{X}^\sigma}$ (Def. 9). Finally, we use the criteria $\text{TRS}^{Y}_{\mathbf{X}^\sigma} > \text{TRS}^{\text{thres}}$ to infer the regulation. Thus, we have three kinds of thresholds (R^{thres}, S^{thres}, and $\text{TRS}^{\text{thres}}$) in our approach. Here, we describe optimizing these threshold values.

First, we test the accuracy of our method on simulated time-series data (Fig. 2b-f) when the R^{thres} and S^{thres} vary. The accuracy is measured by using the F_2 score, and we used 10 values of R^{thres} uniformly sampled from $[0.01, 0.1]$, and 100 values of S^{thres} and $\text{TRS}^{\text{thres}}$ uniformly sampled from $[0.01, 1]$. For each system in the presence of 10% multiplicative noise, we compute TRS using each pair of R^{thres} and S^{thres}. Then, we can obtain the maximum F_2 score by comparing the result from all the possible $\text{TRS}^{\text{thres}}$ values. We repeat this process 10 times and the average of the maximum F_2 score is plotted (Supplementary Fig. 5a). For all the examples, the F_2 score changes greatly when S^{thres} varies. However, the F_2 score is not much affected by the R^{thres}. Thus, in this paper, we fix the value of R^{thres} as 0.05 for 1D and 2D methods, 0.01 for the 3D method, and 0.005 for the 4D method. We decrease R^{thres} as the dimension increases because the average of size($R_{\mathbf{X}^\sigma}$) decreases exponentially as the dimension increases (Remark 3).

Next, with the fixed R^{thres}, we test the accuracy of our approach when the S^{thres} and $\text{TRS}^{\text{thres}}$ vary. For the Kim-Forger model in the presence of 10% multiplicative noise, the F_2 score is computed from each pair of S^{thres} and $\text{TRS}^{\text{thres}}$. We plot all the pairs ($S^{\text{thres}}, \text{TRS}^{\text{thres}}$) which produce high F_2 scores, i.e., $\geq$95% of the maximum F_2 score (Supplementary Fig. 5b). Among these, not every pair of thresholds is optimal. For example, when the thresholds are chosen in the thin region (Supplementary Fig. 5b (i and iii)), then small changes of thresholds might greatly reduce the accuracy. However, when the thresholds are chosen in the thick region (Supplementary Fig. 5b (ii)), then small changes of S^{thres} and $\text{TRS}^{\text{thres}}$ do not reduce the accuracy. Thus, our approach is not sensitive to the value of thresholds when they are chosen in the thick region. The robustness also can be identified by comparing the results when the thresholds are chosen in the thin region and the thick region (Supplementary Fig. 5c). When the S^{thres}

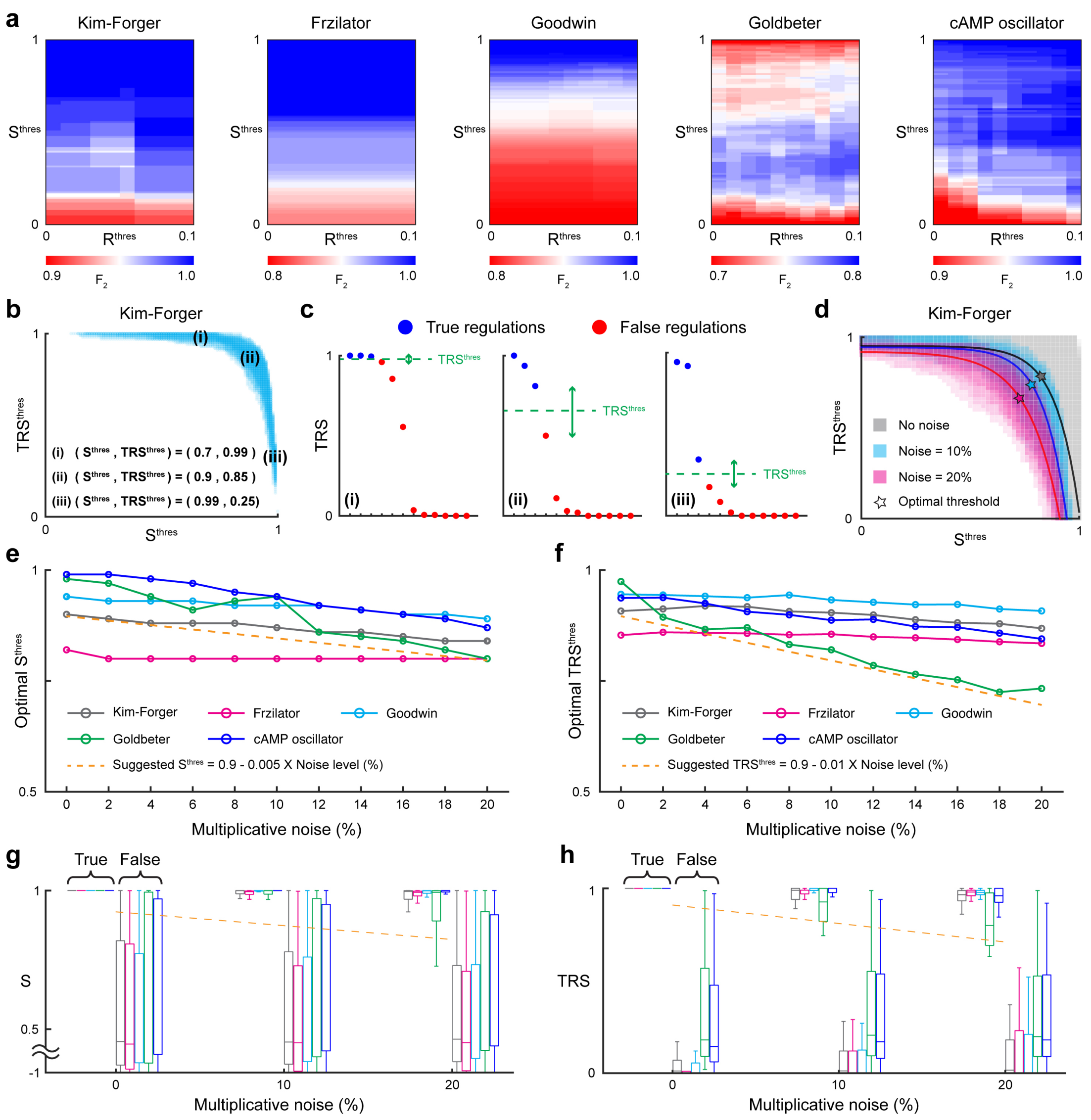

Supplementary Fig. 5. Optimizing the threshold values used in GOBI. **a** For each *in silico* system with the presence of 10% multiplicative noise, we compute the F_2 score when the thresholds for regulation-detection region (R^{thres}) and score (S^{thres}) vary. The F_2 score changes greatly when S^{thres} varies. However, the F_2 score is not much affected by the change of R^{thres}. **b** In the Kim-Forger model, various pairs of S^{thres} and the threshold for TRS ($\text{TRS}^{\text{thres}}$) produce high F_2 scores (i.e., $\geq$95% of maximum F_2 score). Among these pairs, we compare the results when the thresholds are chosen in the thin region (i) (S^{thres} is small) and (iii) (S^{thres} is large) or in the thick region (ii). **c** When S^{thres} is small, a high value of $\text{TRS}^{\text{thres}}$ is needed to distinguish the true and false regulations (left). Conversely, when S^{thres} is large, then a small value of $\text{TRS}^{\text{thres}}$ is needed (right). In both cases, the possible range for the $\text{TRS}^{\text{thres}}$ is narrow. This indicates that the results are sensitive to the value of the thresholds. However, when the thresholds are chosen in the thick region (middle), the result is robust to the value of thresholds. Thus, we choose the thresholds in the middle of the thick region as the optimal thresholds. **d** In the presence of 0, 10, and 20% of multiplicative noise, the pairs of thresholds ($S^{\text{thres}}, \text{TRS}^{\text{thres}}$) which produce high F_2 scores are computed. The values of thresholds decrease as the noise level increases. For each level of noise, we fit the pair of thresholds using an exponential function and find the optimal threshold pair (star) which is the nearest point from $(1, 1)$. The optimal thresholds also decrease as the noise level increases. **e,f** For each *in silico* system, the optimal threshold of S^{thres} (**e**) and $\text{TRS}^{\text{thres}}$ (**f**) decrease as the noise level increases. Based on this tendency, we suggest the guide of optimal thresholds with respect to the level of noise (dashed line). **g** The guide of optimal S^{thres} well distinguishes the regulation-detection score of true and false regulations. **h** Using the guide of optimal S^{thres}, total-regulation scores are computed. The guide of optimal $\text{TRS}^{\text{thres}}$ well distinguishes between the TRS of true and false regulations. Here, for each system, results from 1,000 time-series data are presented as box plots, in which the box bounds the IQR divided by the median, and whiskers extend to a maximum of $1.5 \times$ IQR beyond the box. Source data are provided as a Source Data file.

from the thick region is used, the difference between the TRS for true and false regulations is large (Supplementary Fig. 5c, middle). Thus, we can choose $\text{TRS}^{\text{thres}}$ from a wide range without affecting the results. This indicates that our approach is robust to the choice of thresholds. However, when the S^{thres} from the thin region is used, the possible range of $\text{TRS}^{\text{thres}}$ is narrow and this indicates that our approach becomes sensitive to the thresholds (Supplementary Fig. 5c, left and right). Thus, the optimal threshold is chosen in the middle of the thick region.

In Figure 3, we suggested that the value of S^{thres} and $\text{TRS}^{\text{thres}}$ decreases as the noise level increases. This tendency can be identified by the value of optimal thresholds when the level of noise varies. Here, we plot the pairs $(S^{\text{thres}}, \text{TRS}^{\text{thres}})$ which produce high F_2 scores as the noise level increases (Supplementary Fig. 5d). In the presence of 20% of multiplicative noise (red), the value of thresholds are decreased compared to 10% of multiplicative noise (blue). To quantify this tendency, we approximate the pairs of thresholds using an exponential function, i.e., $\text{TRS}^{\text{thres}} = a \cdot e^{b \cdot S^{\text{thres}}} + c$ where a, b and c are parameters. Note that the fitted line moves to the origin $(0, 0)$ as the noise level increases. We choose the optimal threshold (star) on the fitted line at the nearest point from $(1, 1)$. Then, the values of optimal thresholds decrease as the noise level increases. This tendency is consistent for every *in silico* system (Supplementary Fig. 5e, f). Thus, we suggest the guide of thresholds, which is suitable for every system. Specifically, $S^{\text{thres}} = 0.9 - 0.005 \times (\text{noise level})$ and $\text{TRS}^{\text{thres}} = 0.9 - 0.01 \times (\text{noise level})$ are suggested. However, depending on whether the goal is decreasing false positive or negative predictions, one can adjust the threshold (i.e., increase or decrease the threshold).

IX. DISTINGUISH INDIRECT REGULATION USING COMBINED p-VALUE

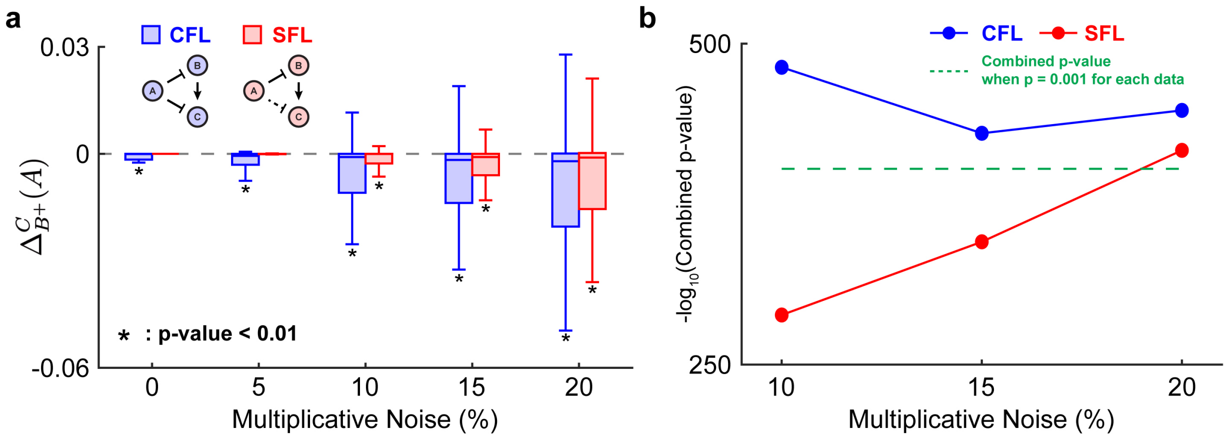

Supplementary Fig. 6. Combined p-value can distinguish between direct and indirect regulations in the presence of noise. **a** In CFL, direct regulation $A \dashv C$ exists. On the other hand, in SFL, only indirect negative regulation from A to C, induced from a regulatory chain $A \dashv B \to C$, exists. In the absence of noise, Δ successfully detects the indirect regulation in SFL as the false positive. Note that $\Delta^C_{B+}(A) = 0$ in SFL whereas $\Delta^C_{B+}(A) \neq 0$ in CFL. This indicates that A does not affect an existing regulation $B \to C$ in SFL. However, in the presence of the $\geq$10% of multiplicative noise, the value of $\Delta^C_{B+}(A)$ often has a negative sign not only in CFL but also in SFL. The negative sign of $\Delta^C_{B+}(A)$ reflects that the new component A negatively affects an existing regulation $B \to C$. This indicates the existence of direct negative regulation from A to C which is indirect regulation in SFL. Here, data are presented as box plots (n = 100), in which the box bounds the IQR divided by the median, and whiskers extend to a maximum of 1.5 × IQR beyond the box. Overall negative signs of $\Delta^Y_{X+}(Z)$ are measured by a one-tailed Wilcoxon signed rank test. Note that $\Delta^C_{B+}(A)$ is significantly negative (i.e., p-value < 0.01) for SFL when either 10, 15, or 20% of multiplicative noise is added. For CFL, $p = 1.24 \times 10^{-13}, 4.26 \times 10^{-10}, 8.28 \times 10^{-9}, 8.38 \times 10^{-5},$ and 1.19×10^{-4} in the presence of $0, 5, 10, 15,$ and 20% of noise, respectively. For SFL, $p = 0.41, 0.23, 1.24 \times 10^{-4}, 9.67 \times 10^{-4},$ and 3.0×10^{-3} in the presence of $0, 5, 10, 15,$ and 20% of noise, respectively. Thus, the Δ test often fails to distinguish between direct and indirect regulations in the presence of noise. **b** To prevent such false positive prediction, we compute $S^C_{A^-_{\text{shuffled}}B+}$ with surrogate time series A and compare with the original S^C_{A-B+}. In the presence of direct regulation (CFL), but not indirect regulation (SFL), $S^C_{A^-_{\text{shuffled}}B+}$ is significantly smaller than S^C_{A-B+} and the significance (i.e., p-value) is quantified by a one-tailed Z test. Those p-values from all the data are combined into one test statistic (χ^2) using Fisher's method. The combined p-value successfully distinguishes between direct and indirect regulation from CFL and SFL in the presence of the $10, 15$% of multiplicative noise. We set the critical value of the significance of Fisher's method by combining p-value $= 0.001$ for all the data. Source data are provided as a Source Data file.

In the presence of noise, the Δ test often fails to distinguish between direct and indirect regulations from A to C in CFL and SFL (Fig. 3f, g). Specifically, in CFL and SFL with the presence of $\geq$ 10% multiplicative noise, $\Delta^C_{B+}(A) < 0$ for most cases (Supplementary Fig. 6a). This reflects that there is negative direct regulation from A to C not only in CFL but also in SFL. To check if this regulation is direct or indirect, we perform the surrogate test. The combined p-value successfully distinguishes between direct and indirect regulation in the presence of 10% and 15% of multiplicative noise (Supplementary Fig. 6b).

X. THE ROBUSTNESS OF GOBI TO VARIOUS TYPES OF NOISE

In the main text, we considered multiplicative noise as the measurement noise (Fig. 3). Here, we investigate the robustness of GOBI by introducing different noise models, such as additive noise, colored noise, and dynamical noise.

First, let $X = \{X(t_1), X(t_2), \cdots, X(t_N)\}$ denote the original time series that are normalized after being simulated from the ODE. To generate the noisy time series with the multiplicative noise level of m%, we randomly sample a noise $\varepsilon = \{\varepsilon_1, \varepsilon_2, \cdots, \varepsilon_N\}$ from a normal distribution with a mean of 0 and a standard deviation of 1 (i.e., $\varepsilon_i \sim \mathcal{N}(0, 1)$, where $i = 1, 2, \cdots, N$). Then, we multiply the noise ε proportional to the noise level and add it to the original time series to obtain the noisy time series as follows:

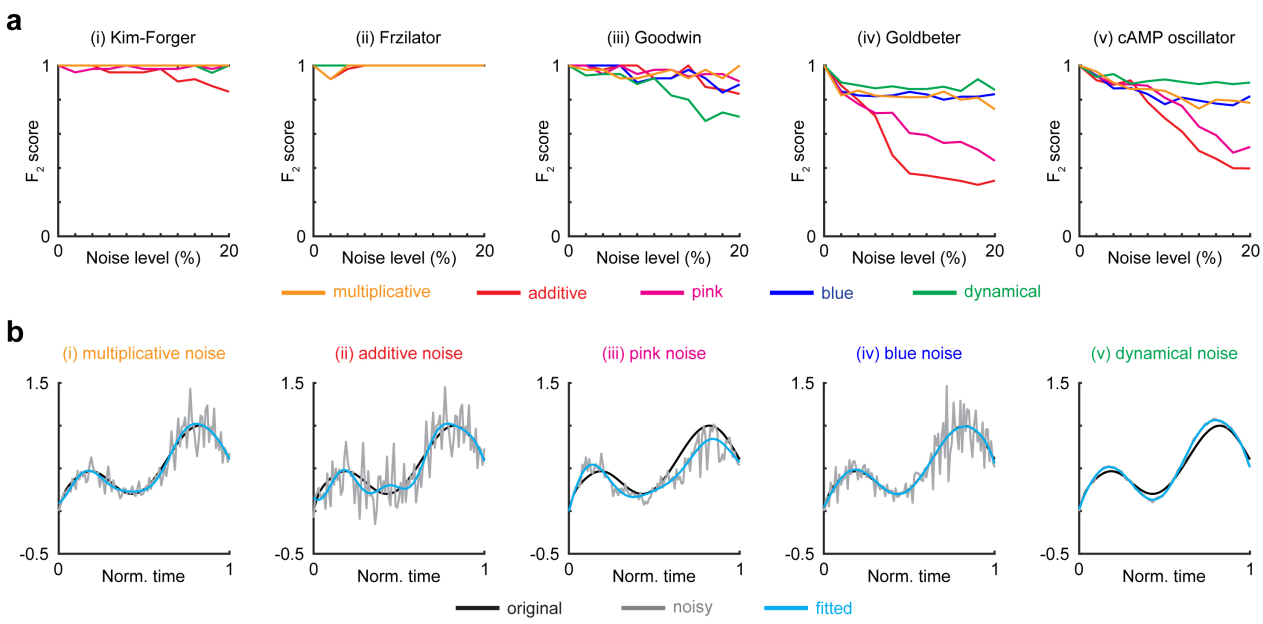

Supplementary Fig. 7. The robustness of GOBI to various types of noise. **a** The robustness of GOBI is tested under different types of noise added to time-series data simulated using various ODE models: Kim-Forger model (**a** (i)), Frzilator (**a** (ii)), 4-state Goodwin oscillator (**a** (iii)), Goldbeter model (**a** (iv)), and cAMP oscillator (**a** (v)). For each time-series data, five different types of noise, including multiplicative noise, additive noise, pink noise, blue noise, and dynamical noise, are added with noise levels varying from 0 to 20%, respectively. For each noisy time-series data set, GOBI infers a network structure and the F_2 score is calculated. This process is repeated ten times and the mean F_2 score is shown for each system, noise type, and noise level (**a** (i)-(v)). Here, each data set consists of 100 time series simulated with different initial conditions. **b** The original time series is simulated from cAMP oscillator (black line), and a 20% level of noise is added (grey line) for each type of noise: multiplicative noise (**b** (i)), additive noise (**b** (ii)), pink noise (**b** (iii)), blue noise (**b** (iv)), and dynamical noise (**b** (v)). Then, the Fourier method is applied to fit the noisy time series (blue line). Source data are provided as a Source Data file.

$$X_{\text{multiplicative noise}} := \left\{X(t_1)\cdot(1+\frac{m}{100}\cdot\varepsilon_1), X(t_2)\cdot(1+\frac{m}{100}\cdot\varepsilon_2), \cdots, X(t_N)\cdot(1+\frac{m}{100}\cdot\varepsilon_N)\right\}.$$

Next, we generate noisy time series with additive noise. Following a procedure used for generating time series with multiplicative noise, we randomly sample the noise ε from a normal distribution $\mathcal{N}(0,1)$. Then, we add the noise to the original time series proportional to the noise level as follows:

$$X_{\text{additive noise}} := \left\{X(t_1)+\frac{m}{100}\cdot\varepsilon_1, X(t_2)+\frac{m}{100}\cdot\varepsilon_2, \cdots, X(t_N)+\frac{m}{100}\cdot\varepsilon_N\right\}.$$

Third, we generate noisy time series with colored noise, utilizing two representative types: pink noise and blue noise. Pink noise is characterized by strong power at low frequency, while blue noise is characterized by strong power at high frequency. We sample pink noise $\varepsilon^{\text{pink}} = \{\varepsilon_1^{\text{pink}}, \varepsilon_2^{\text{pink}}, \cdots, \varepsilon_N^{\text{pink}}\}$ and blue noise $\varepsilon^{\text{blue}} = \{\varepsilon_1^{\text{blue}}, \varepsilon_2^{\text{blue}}, \cdots, \varepsilon_N^{\text{blue}}\}$ using the MATLAB function 'dsp.ColoredNoise'. Then, we generate noisy time series with a colored noise level of m% as follows:

$$\begin{aligned} X_{\text{pink noise}} &:= \left\{X(t_1)\cdot(1+\frac{m}{100}\cdot\varepsilon_1^{\text{pink}}), X(t_2)\cdot(1+\frac{m}{100}\cdot\varepsilon_2^{\text{pink}}), \cdots, X(t_N)\cdot(1+\frac{m}{100}\cdot\varepsilon_N^{\text{pink}})\right\} \text{ and} \\ X_{\text{blue noise}} &:= \left\{X(t_1)\cdot(1+\frac{m}{100}\cdot\varepsilon_1^{\text{blue}}), X(t_2)\cdot(1+\frac{m}{100}\cdot\varepsilon_2^{\text{blue}}), \cdots, X(t_N)\cdot(1+\frac{m}{100}\cdot\varepsilon_N^{\text{blue}})\right\}. \end{aligned}$$

Until now, we have only taken into account the effects of measurement noise. To make GOBI more widely applicable, we also need to consider the influence of dynamical noise. To generate noisy time series with dynamical noise, we add a noise term to the ODE. Specifically, let the dynamic of Y be given as follows:

$$\frac{dY}{dt} = f(\mathbf{X}).$$

Then, the noisy time series $Y_{\text{dynamical noise}}$ with the dynamical noise level of $m\%$ is simulated from the following ODE:

$$\frac{dY_{\text{dynamical noise}}}{dt} = f(\mathbf{X}) + Y_{\text{mean}} \cdot \frac{m}{100} \cdot \varepsilon,$$

where Y_{mean} is the mean of the original time series Y, and ε is randomly sampled from a normal distribution $\mathcal{N}(0, 1)$.

Now, we illustrate the robustness of GOBI to different types of noise. Using various ODE models (Fig. 2b-f), we simulate 100 time series from randomly selected initial conditions which lie in the range of the original limit cycle. For each time series, we introduce five types of noise, with noise levels varying from 0 to 20%, as previously described. From each noisy time-series data set, we use the Fourier method (i.e., using the MATLAB function 'fit' with the 'fourier4' option) to obtain fitted time-series data, and then apply GOBI to infer a network structure. Then, we calculate the F_2 score for each system, noise type, and noise level. This process is repeated ten times, and the mean F_2 scores are presented (Supplementary Fig. 7a).

For simple systems, such as the Kim-Forger model, Frzilator, and 4-state Goodwin oscillator, GOBI successfully infers regulatory networks regardless of the type of noise in the simulated time-series data (Supplementary Fig. 7a (i)-(iii)). However, for more complex systems that include 2D regulations, such as the Goldbeter model and cAMP oscillator, GOBI exhibits varying levels of robustness under different types of noise (Supplementary Fig. 7a (iv) and (v)). Specifically, the performance of GOBI is greatly affected by additive noise and pink noise compared to other types of noise. Additive noise is added independently of the original signal's value, whereas multiplicative noise is added as a proportion of the signal. Thus, additive noise has a greater impact on the signal's overall shape and magnitude than multiplicative noise (Supplementary Fig. 7b (i) and (ii)). Additionally, pink noise has more power at low frequencies than blue noise, making it more likely to affect the shape of time series (Supplementary Fig. 7b (iii) and (iv)). Taken together, noise types that significantly affect the shape of trajectories, such as additive noise and pink noise, can result in the decreased performance of GOBI because the algorithm uses time series shape information for inference.

XI. DESCRIPTION OF THE EXPERIMENTAL TIME-SERIES DATA

1. *Prey-predator system*

The population time-series data of prey, *Paramecium* and predator, *Didinium* for 30 days (Fig. 4a) is taken from [10]. Because their period is about five days, we cut the data for every five days to generate seven different time-series data sets. Each data set is interpolated using the MATLAB function 'spline' before applying our method.

2. *Genetic oscillator*

The multiple time series of a synthetic oscillator (Fig. 4b), which consists of TetR and σ^{28}, are taken from [11]. Because their periods vary as TetR is perturbed, we cut the time series with different scales (periods) to generate 15 time series data sets. Then, each data set is interpolated using the MATLAB function 'spline' before applying our method.

3. *Repressilator*

The experimental data sets of the repressilator (Fig. 4c) are taken from [12]. Since the number of cycles in the data is low (< 5) to generate enough multiple time series, each time series is cut using the moving-window technique to generate multiple time series data sets with various phases. Then, each data set is interpolated using the MATLAB function 'spline' before applying our method.

4. *Estradiol data set*

The estradiol data sets (Fig. 4d) are taken from [13]. Each time series is cut using the moving-window technique. Then, each time series is interpolated using the MATLAB function 'spline' before applying our method.

5. *Air pollution and cardiovascular disease*

The time-series data of air pollutants and cardiovascular disease occurrence in the city of Hong Kong from 1994 to 1997 (Fig. 4e) is taken from [14]. We use the time series of 1,000 days from March 1995 to November 1997 to avoid the effect of a sudden addition of hospital beds in early 1995, as done in the previous study [15]. The time series of disease admissions is smoothed using a simple moving average with a window width of seven days to avoid the effect of days of the week. Then, each time series is cut with a window size of one year and an overlap of 11 months (i.e., move the window for a month) to generate 23 data sets. Because of a high noise level, we smooth the time series using the MATLAB function 'fit' with an option 'fourier2' before applying our method.

XII. INFERENCE RESULTS FROM EXPERIMENTAL DATA SET

a

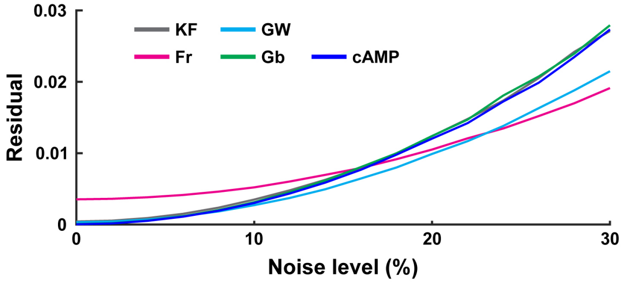

b

System	Residual	Noise level
prey-predator	3.7e-4	~5%
genetic oscillator	1.2e-3	~8%
repressilator	1.1e-3	~8%
Estradiol	3.7e-4	~5%
Cardio	1.8e-2	~30%

Supplementary Fig. 8. Approximate noise level of experimental data using residual. **a** For each *in silico* system, we compute the mean square of the residual between noisy and fitted time series when the noise level varies. Here, fitted time series are obtained by using the MATLAB function 'fit' with an option 'fourier4'. The mean square of the residual is averaged over all components in the system and we simply called it 'residual'. The value of the residual increases as the noise level increases and their tendency is similar among the systems. **b** Using this tendency, we can approximate the noise level of experimental data. For each system that we use (prey-predator, genetic oscillator, repressilator, estradiol data set and air pollutants, and cardiovascular disease), experimental time-series data are interpolated using the MATLAB function 'fit' with an option 'fourier4'. Then, we compute the residuals and approximate their noise level. Source data are provided as a Source Data file.

Here, we describe detailed inference results of GOBI on experimental data sets. Before applying our method, the noise level of each data set is approximated to determine the thresholds for the regulation-detection score and total-regulation score (Supplementary Fig. 8).

First, we apply our method to the prey-predator system. Since this system consists of two components, we only consider the criteria of TRS for 1D regulation to infer the regulation. TRS is calculated for each set of cause and target (Supplementary Fig. 9a rows) and for each regulation type (Supplementary Fig. 9a columns). The criteria $\text{TRS}^{Y}_{\mathbf{X}^{\sigma}Y^{-}} > \text{TRS}^{\text{thres}}$ infers two direct regulations, $P \rightarrow D$ and $D \dashv P$.

Similarly, for the genetic oscillator, the criteria of TRS for 1D regulation infers two direct regulations, TetR $\dashv \sigma^{28}$ and $\sigma^{28} \rightarrow$ TetR (Supplementary Fig. 9b (i)). Then, we check the 2D regulations including self-regulation. Two 2D regulations, $\substack{\text{TetR}\dashv \\ \sigma^{28}\rightarrow}\text{TetR}$ and $\substack{\text{TetR}\dashv \\ \sigma^{28}\dashv}\sigma^{28}$, are inferred using the criteria $\text{TRS}^{Y}_{\mathbf{X}^{\sigma}Y^{-}} > \text{TRS}^{\text{thres}}$ (Supplementary Fig. 9b (ii)). Both regulations pass the Δ test, and we do not perform the surrogate test as there are no possible indirect regulations in the two-component system. This indicates that assumptions incorporating prior information (such as the types of self-regulation) are unnecessary when sufficient data is present.

Next, for the repressilator, our 1D framework infers three negative 1D regulations, LacI $\dashv \lambda$cl, λcl $\dashv$ TetR, and TetR $\dashv$ LacI (Supplementary Fig. 9c (i)). Then, we need to check the 2D regulation as well since the system consists of three components. Three 2D regulations are inferred using the criteria $\text{TRS}^{Y}_{\mathbf{X}^{\sigma}Y^{-}} > \text{TRS}^{\text{thres}}$, i.e., $\substack{\lambda\text{cl}\dashv \\ \text{LacI}\rightarrow}\text{TetR}$, $\substack{\lambda\text{cl}\rightarrow \\ \text{TetR}\dashv}\text{LacI}$, and $\substack{\text{LacI}\dashv \\ \text{TetR}\rightarrow}\lambda\text{cl}$ (Supplementary Fig. 9c (ii)). Red boxes indicate that the regulation-detection region is

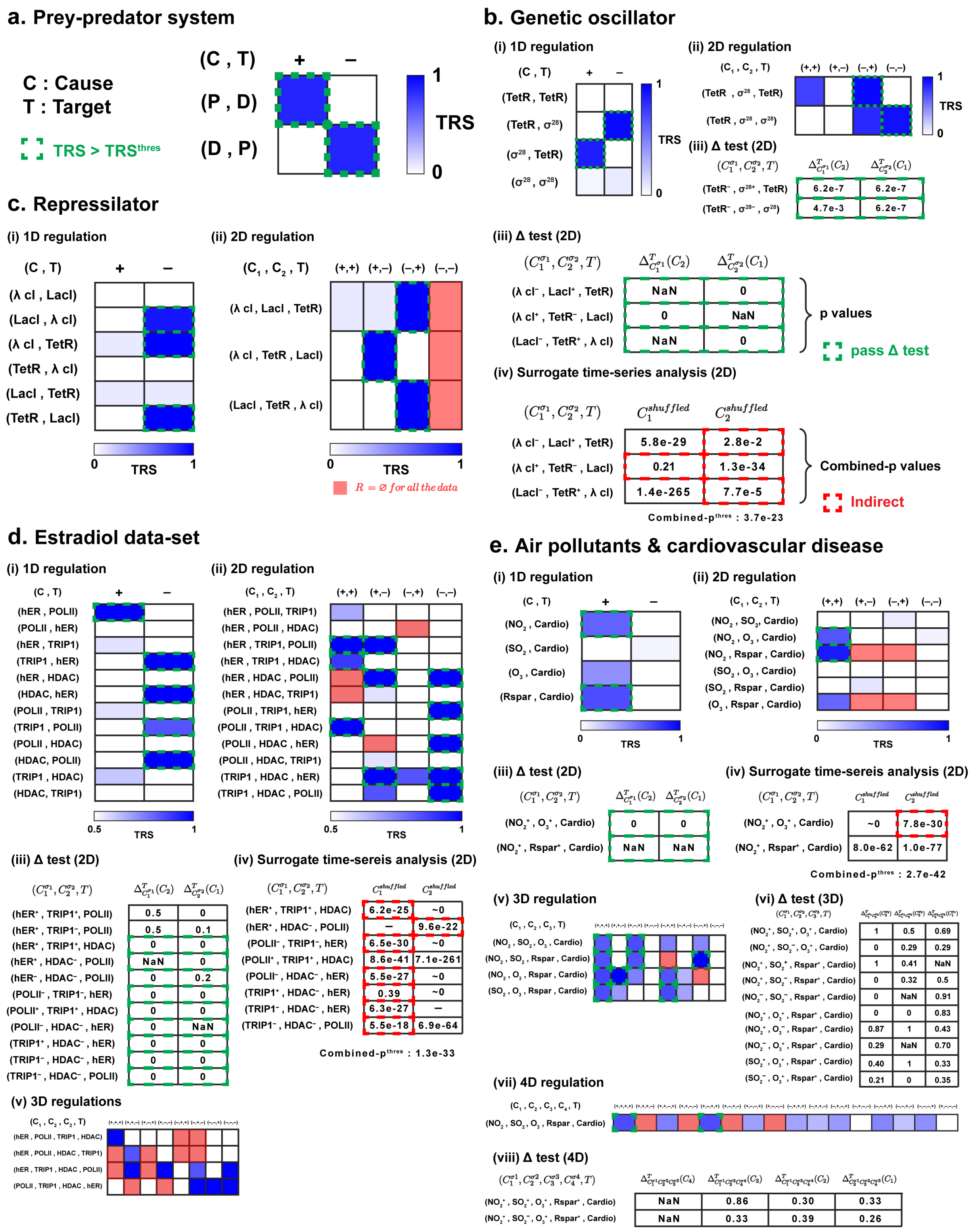

Supplementary Fig. 9. Inferring regulation using total-regulation score, Δ test, and surrogate test from experimental time-series data: prey-predator system (**a**), genetic oscillator (**b**), repressilator (**c**), estradiol data set (**d**), and cardiovascular disease caused by air pollutants (**e**). For the Δ test, p-values from Wilcoxon signed ranked test are presented for the genetic oscillator (**b**) and the ratio of positive sign are presented for the others (**a**, **c**-**e**). For the surrogate test, p-values from one-tailed Z test are combined using Fisher's method. Source data are provided as a Source Data file.

empty (i.e., size$(R_{\mathbf{X}^\sigma Y^-}) < R^{\text{thres}}$) for every data set. Next, for inferred 2D regulations, we compute the regulation-delta function, and the ratio of data which does not satisfy the criteria for the Δ test is listed (Supplementary Fig. 9c (iii)). Thus, the regulations pass the Δ test only when the ratio is zero (Supplementary Fig. 9c (iii) green box). Because of the empty regions, Δ is not calculable for some cases (Supplementary Fig. 9c (iii) NaN in the table) and thus no false positive predictions are detected. Then, the surrogate test distinguishes between direct and indirect regulations. For instance, $S^{\text{TetR}}_{\lambda\,\text{cl}^-\text{LacI}^+}$ is significantly larger than $S^{\text{TetR}}_{\lambda\,\text{cl}^-_{\text{shuffled}}\text{LacI}^+}$, which represents the direct regulation λ cl $\dashv$ TetR, whereas $S^{\text{TetR}}_{\lambda\,\text{cl}^-\text{LacI}^+}$ is not significantly larger than $S^{\text{TetR}}_{\lambda\,\text{cl}^-\text{LacI}^+_{\text{shuffled}}}$, which represents the indirect regulation LacI $\rightarrow$ TetR (red dashed box) (Supplementary Fig. 9c (iv)).

We next apply our method to the estradiol data set (Supplementary Fig. 9d). Using 1D and 2D TRS, five 1D regulations and eleven 2D regulations are inferred using the criteria $\text{TRS}^{Y}_{\mathbf{X}^\sigma Y^-} > \text{TRS}^{\text{thres}}$ (Supplementary Fig. 9d (i) and (ii)). We exclude inferred 1D regulations because they share the same target. Then, the Δ test removes three false positive predictions (Supplementary Fig. 9d (iii)). Among inferred regulations, we identify the regulations which have the potential to be indirect, then perform the surrogate test ('$-$' in the table indicates no potential to be indirect regulation) (Supplementary Fig. 9d (iv)). Finally, we identify the direct regulation hER $\rightarrow$ POLII, HDAC $\dashv$ hER, and $\substack{\text{POLII}\rightarrow \\ \text{TRIP1}\rightarrow}$ HDAC. Due to the limited amount of data, TRS is not calculable for half of the possible 3D regulation types (Supplementary Fig. 9d (iv)). Thus, we are not able to further infer 3D regulations.

Finally, for the air pollutants and cardiovascular disease data, our 1D method infers $NO_2 \rightarrow Cardio$ and $Rspar \rightarrow Cardio$ (Supplementary Fig. 9e (i)). We exclude them because they share the same target. Next, the criteria of TRS for 2D regulation infers $\substack{NO_2\rightarrow \\ O_3\rightarrow} Cardio$ and $\substack{NO_2\rightarrow \\ Rspar\rightarrow} Cardio$ (Supplementary Fig. 9e (ii)). Δ test fails to distinguish false positive regulations (Supplementary Fig. 9e (iii)), but the surrogate test finds indirect regulation $O_3 \rightarrow Cardio$ (Supplementary Fig. 9e (iv)). Here, we consider that every inferred regulation has the potential to be indirect because data on other factors, such as temperature, humidity, and wind speed, are not completely available, which can be the common causes of some pollutants and induce indirect effects. Thus, for the 2D regulation, only $\substack{NO_2\rightarrow \\ Rspar\rightarrow} Cardio$ is inferred. Also, several 3D regulations are inferred by the criteria of TRS for 3D regulation (Supplementary Fig. 9e (v)), but none of them pass the criteria of the Δ test (Supplementary Fig. 9e (vi)). Similarly, none of the 4D regulations are inferred from the criteria of TRS and Δ test (Supplementary Fig. 9e (vii) and (viii)).

XIII. THE ACCURACY OF GOBI ON EXPERIMENTAL DATA AT DIFFERENT SAMPLING RATES

Here, we investigate how the accuracy of GOBI and model-free methods (GC, CCM, and PCM) is affected by the sampling rates of experimental time-series data obtained from systems with known regulatory networks (Fig. 5a-b). After varying the sampling rates of each time-series data, we apply GOBI and model-free methods, including GC, CCM, and PCM, and compute the F_2 scores of the inference results (Supplementary Fig. 10b). Model-free methods infer a lot of false positive predictions regardless of the sampling rate because they often misidentify synchrony for causality (Supplementary Fig. 10c). While PCM can infer the true network structure (i.e., two independent feedback loops) of the prey-predator system merged with the genetic oscillator from the original experimental data, its accuracy drops dramatically when the sampling rate is reduced (Supplementary Fig. 10b (i)). However, GOBI successfully infers true network structures even when the sampling rate is halved (Supplementary Fig. 10b-c). When the sampling rate is reduced by a quarter, which significantly changes the shape of the time series (Supplementary Fig. 10a (i)-(ii) bottom), the accuracy of GOBI also decreases, but it is still comparable to that of model-free methods (Supplementary Fig. 10b-c). This indicates that GOBI is robust to changes in the sampling rate as long as the shape of the time series is preserved.

XIV. EXTENSION TO TEMPORAL-STRUCTURED MODEL INCLUDING NON-MONOTONIC REGULATION

Here, we illustrate an example of how GOBI can be applied to a temporal-structured system that includes non-monotonic regulation. To construct a temporal-structured regulation from A to C (Supplementary Fig. 11a (i)), the values of parameters (α, β, and γ) are varied over time (Supplementary Fig. 11a (ii)), resulting in different types of regulation from A to C: positive regulation in the first quarter, non-monotonic regulation in the second quarter, negative regulation in the third quarter, and the absence of regulation in the last quarter. We simulate 100 time-series data of C from different input signals A and B (Supplementary Fig 11b). The time series of input signals are constructed by randomly selecting 200 points from $[0, 1]$ over the time domain and connecting them with the 'spline' method.

Next, we use a moving-window technique with a window size of 5 and an overlapping ratio of 50% to segment the

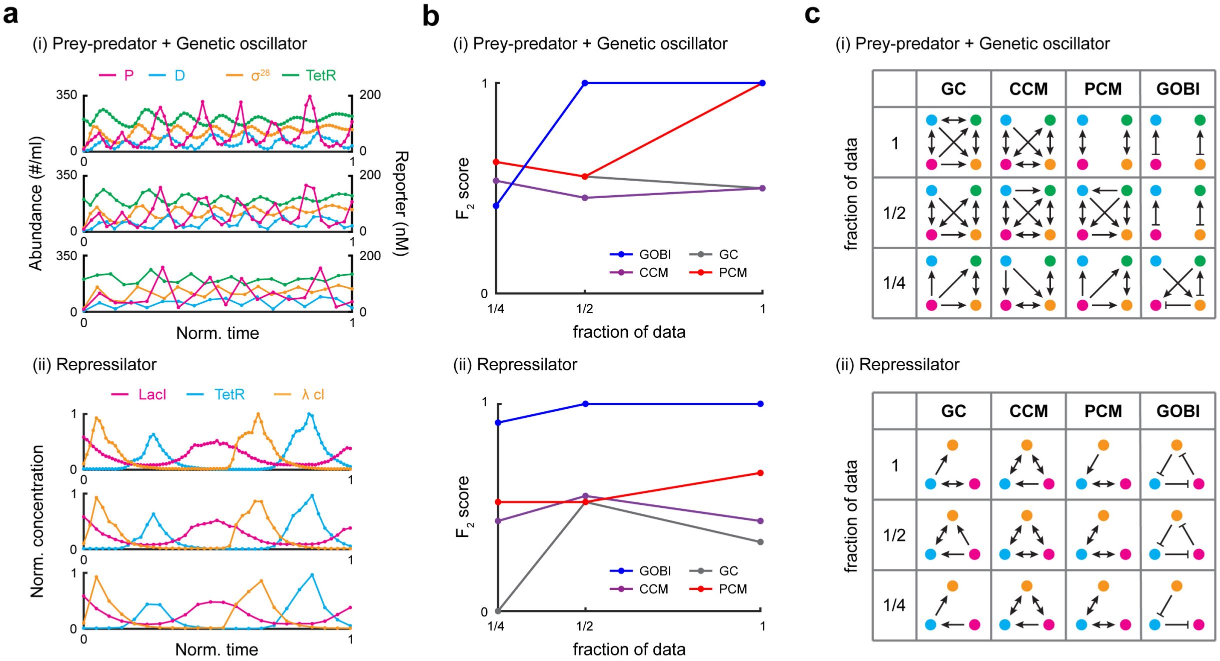

Supplementary Fig. 10. Accuracy of GOBI on experimental data at different sampling rates. **a** Time-series data from two different systems are used: the prey-predator system merged with the genetic oscillator (**a** (i)) and the repressilator (**a** (ii)). For each system, time-series data are obtained at different sampling rates: using all time points (**a** (i)-(ii) top), one for every two adjacent time points (**a** (i)-(ii) middle), and one for every four adjacent time points (**a** (i)-(ii) bottom). **b** Using time-series data with varying sampling rates, the accuracy of GOBI and model-free methods (GC, CCM, and PCM) is tested and F_2 scores are calculated. **c** For each system, the inferred network structures at different sampling rates are illustrated. Source data are provided as a Source Data file.

time-series data of A, B, and C. For each window, regulation-detection scores for positive and negative regulation (S^C_{A+C-} and S^C_{A-C-}) are computed from the 100 time series (Supplementary Fig. 11c). The criterion $S^C_{A+C-} = 1$ ($S^C_{A-C-} = 1$) is satisfied in the presence of positive (negative) regulation (Supplementary Fig. 11c, the first and third quarter). However, in the presence of non-monotonic regulation or the absence of regulation, neither criterion is satisfied, indicating that the regulation-detection score cannot distinguish between these two cases (Supplementary Fig. 11c, the second and last quarter). In particular, when the strength of both positive and negative regulation is similar, the regulation-detection scores are around zero (the middle of the second quarter in Supplementary Fig. 11c, which is similar to the case of the absence of regulation.

On the other hand, the regulation-detection functions instead of their normalized integrals (i.e., regulation-detection scores) differ between non-monotonic regulation and the absence of regulation. To distinguish between these cases, we utilize the regulation-detection functions I^C_{A+C-} and I^C_{A-C-}, which correspond to positive and negative regulation from A to C, respectively. These functions, I^C_{A+C-} and I^C_{A-C-}, are defined on the regulation-detection regions R_{A+C-} (bottom-right triangle, Supplementary Fig. 11d) and R_{A-C-} (top-left triangle, Supplementary Fig. 11d), respectively. We compute I^C_{A+C-} and I^C_{A-C-} for each time series window using all available data. As expected, the presence of positive and negative regulations resulted in positive values for I^C_{A+C-} and I^C_{A-C-} on whole their domains, respectively (Supplementary Fig. 11e (i) and (iii)). On the other hand, in the presence of non-monotonic regulation or the absence of regulation, the regulation-detection functions have both positive and negative values (Supplementary Fig. 11e (ii) and (iv)). However, the patterns of the mixture of positive and negative values are completely different between non-monotonic regulation (Supplementary Fig. 11e (ii)) and the absence of regulation (Supplementary Fig. 11e (iv)). Specifically, in the absence of regulation, the sign of the regulation-detection function varies inconsistently across the time-series data, resulting in a completely mixed sign of the regulation-detection function (Supplementary Fig. 11e (iv)). In contrast, in the presence of non-monotonic regulation, the sign of the regulation-detection function is consistent across the time-series data (Supplementary Fig. 11e (ii)). Taken together, I^C_{A+C-} and I^C_{A-C-} are consistently positive or negative at the specific values of $(A(t), A(t^*))$ in the presence of non-monotonic regulation, but not in the absence of regulation.

We quantify this consistency to distinguish between non-monotonic regulation and the absence of regulation. We first divide the regulation-detection regions (R_{A+C-} and R_{A-C-}) into triangles of equal shape (Supplementary Fig. 11f

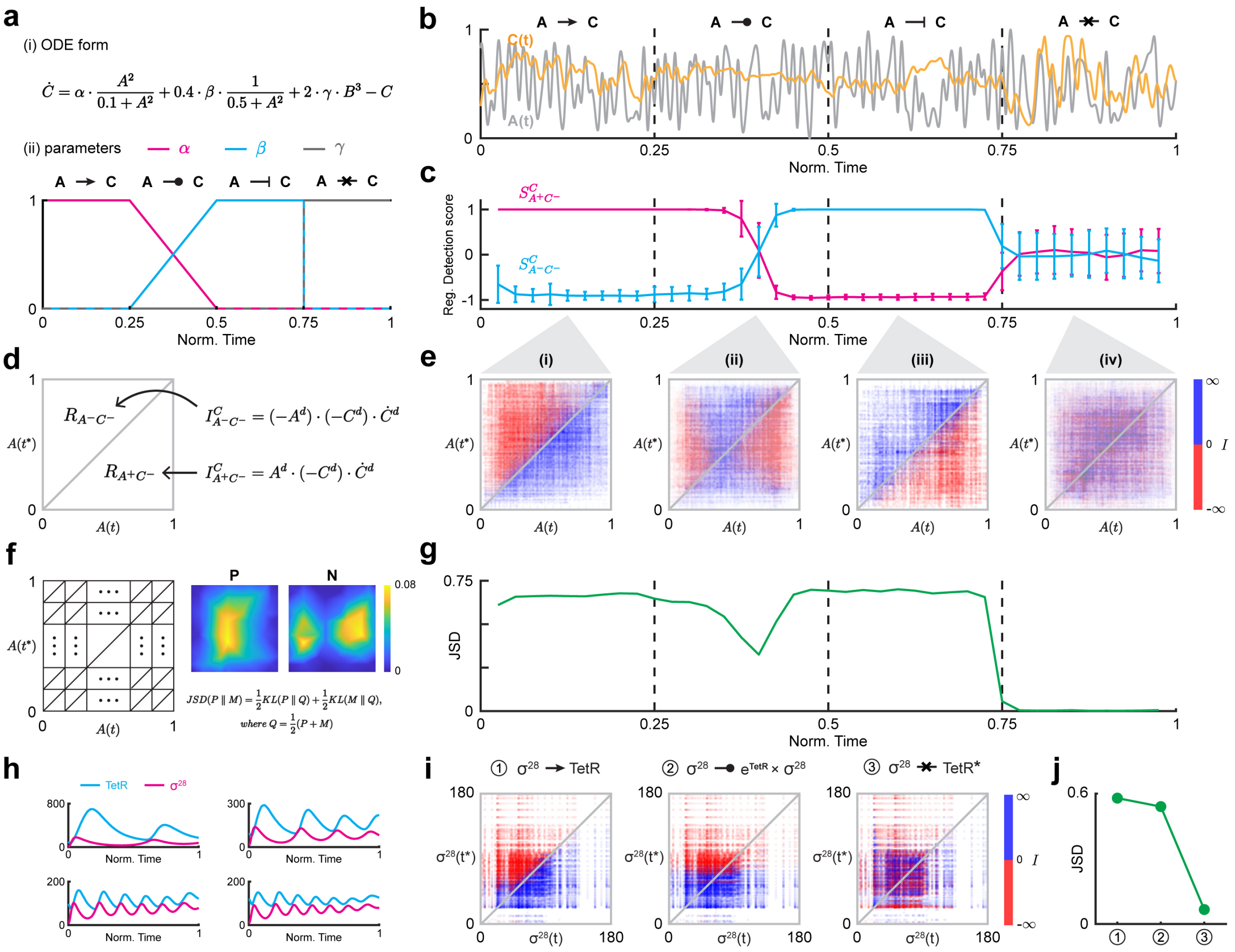

Supplementary Fig. 11. Extended framework of GOBI to distinguish non-monotonic regulation from the absence of regulation. **a** As described by the ODE (**a** (i)), the time-varying values of parameters (α, β, and γ) (**a** (ii)) result in different types of regulation from A to C: positive regulation in the first quarter, non-monotonic regulation in the second quarter, negative regulation in the third quarter, and the absence of regulation in the last quarter. **b** With the ODE describing the network (**a** (i)), 100 time series are simulated from different initial conditions and one of them is shown. **c** Regulation-detection scores are computed for positive and negative regulations (S^C_{A+C-} and S^C_{A-C-}), and the mean value and standard deviation over 100 time-series data are shown. The criterion $S^C_{A+C-} = 1$ ($S^C_{A-C-} = 1$) is satisfied in the presence of positive (negative) regulation, whereas neither is satisfied in the presence of non-monotonic regulation or the absence of regulation. **d-e** Non-monotonic regulation and the absence of regulation can be distinguished by comparing regulation-detection functions (I^C_{A+C-} and I^C_{A-C-}). **d** I^C_{A+C-} and I^C_{A-C-} are defined on the regulation-detection regions R_{A+C-} (bottom-right triangle) and R_{A-C-} (top-left triangle), respectively. **e** The regulation-detection functions, computed from 100 time-series data for each regulation-detection region, are shown with the color indicating its sign. I^C_{A+C-} and I^C_{A-C-} are always positive in the presence of positive and negative regulation (**e** (i) bottom-right triangle and (iii) top-left triangle), respectively. On the other hand, in the presence of non-monotonic regulation and the absence of regulation, I^C_{A+C-} and I^C_{A-C-} have both positive and negative values (**e** (ii) and (iv)). While the regulation-detection functions are always positive or negative at the specific region across all the time-series data for the non-monotonic regulation (**e** (ii)), the region where regulation-detection functions are positive or negative keeps changing across the data for the absence of regulation (**e** (iv)). **f** To quantify this consistency, regulation-detection regions are triangularized (**f** left). Next, the approximated probability distribution of the positive values (**f** right, P) and the negative values (**f** right, N) of I^C_{A+C-} and I^C_{A-C-} are obtained. Then, the Jensen-Shannon Divergence (JSD) is used to measure the similarity between two probability distributions (**f** bottom-right). Here, the 32 number of partitions are used. Also, to illustrate the P and N, the results of regulation-detection functions on the window in the presence of non-monotonic regulation are used (**e** (ii)). **g** The JSD is higher in the presence of non-monotonic regulation than in the absence of regulation. **h-j** This strategy is applied to the time-series data of the genetic oscillator (**h**) by comparing the regulation-detection function in the presence of positive regulation (**i** ①), non-monotonic regulation (**i** ②), and the absence of regulation (**i** ③). JSD is higher in the presence of non-monotone regulation than in the absence of regulation (**j**). Source data are provided as a Source Data file.

left). Then, we count the number of positive values of $I^{C}_{A^+C^-}$ and $I^{C}_{A^-C^-}$ at each triangle, and normalize this count by the total number of positive values to approximate the probability distribution of positive values (Supplementary Fig. 11f right, P). Similarly, we approximate the probability distribution of negative values (Supplementary Fig. 11f right, N). Here, we use the results of regulation-detection functions on the window in the presence of non-monotonic regulation as an example (Supplementary Fig. 11e (ii)). Finally, we quantify the similarity between the probability distribution of positive and negative values (P and N) using the Jensen-Shannon Divergence (JSD) (Supplementary Fig. 11f bottom-right):

$$JSD(P\|N) = \frac{1}{2}KL(P\|Q) + \frac{1}{2}KL(N\|Q), \text{ where } Q := \frac{1}{2}(P+N).$$

A high (low) value of JSD indicates that the regulation-detection function has consistent (inconsistent) sign throughout the region. The similarity, measured by JSD, is much higher when non-monotonic regulation is present compared to the absence of regulation (Supplementary Fig. 11g). This suggests that the consistency test based on JSD can be used to distinguish between non-monotonic regulation and the absence of regulation.

We next investigate whether the proposed consistency test can distinguish between non-monotonic regulation and the absence of regulation when experimental data is given. Specifically, among the eight different time-series data of the genetic oscillator (Fig. 4b), we use four time series with a similar range of σ^{28} (Supplementary Fig. 11h). Using these data, we test the consistency of the signs of regulation-detection functions for three types of regulations: positive regulation of σ^{28} for TetR, non-monotonic regulation of σ^{28} for $\mathrm{e}^{\mathrm{TetR}} \times \sigma^{28}$, and the absence of regulation. The regulation of σ^{28} for $\mathrm{e}^{\mathrm{TetR}} \times \sigma^{28}$ is non-monotonic due to the positive regulation from σ^{28} to TetR and negative self-regulation of σ^{28}. Furthermore, we investigate the regulation of σ^{28} for TetR* for the absence of regulation, where TetR* is measured under different conditions than σ^{28}. For the positive regulation, $I^{\mathrm{TetR}}_{\sigma^{28+}\mathrm{TetR}^-}$ is always positive (Supplementary Fig. 11i ①, bottom-right triangle). For the non-monotonic regulation, $I^{\mathrm{e}^{\mathrm{TetR}}\times\sigma^{28}}_{\sigma^{28-}\mathrm{TetR}^-}$ and $I^{\mathrm{e}^{\mathrm{TetR}}\times\sigma^{28}}_{\sigma^{28+}\mathrm{TetR}^-}$ have both positive and negative values, while their signs are consistent across the time-series data (Supplementary Fig. 11i ②). On the other hand, in the absence of regulation, the sign of the regulation-detection function varies across the data (Supplementary Fig. 11i ③). Consequently, JSD is much higher for non-monotonic regulation than for the absence of regulation (Supplementary Fig. 11j ② and ③), indicating that the consistency test can distinguish between non-monotonic regulation and the absence of regulation.

XV. EXTENSION TO INFER TIME-DELAYED CAUSAL INTERACTIONS

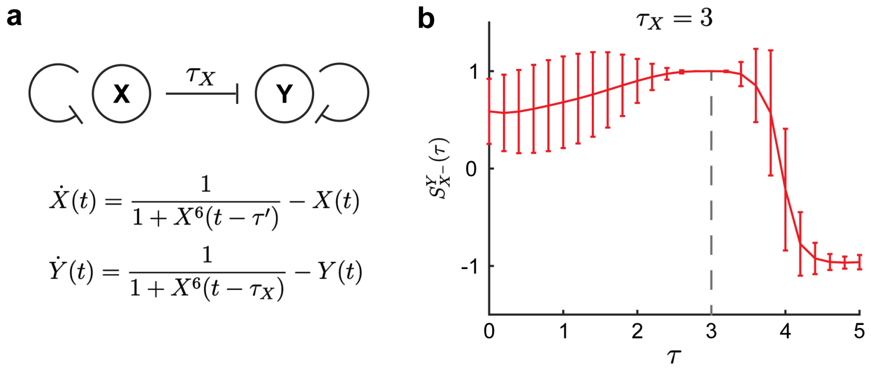

Supplementary Fig. 12. Inferring delayed regulation using an extended regulation-detection score. **a** The network motif contains delayed negative regulation from X to Y (**a** top). The network is described by a delay differential equation, with $\tau' = 5$ and $\tau_X = 3$ as time delays (**a** bottom). Various time series are simulated from different initial conditions, specifically $Y(0) = 1$ and $X(t) = \sin t + \frac{\pi}{10} j$ on $t \in [-5, 0]$ where $j = 1, 2, \cdots, 20$. **b** $S^Y_{X^-}(\tau)$ is computed for each time-series data with different values of τ, varying from 0 to 5. The mean value and standard deviation of $S^Y_{X^-}(\tau)$ over 20 time-series data are shown. Source data are provided as a Source Data file.

When X positively regulates Y with time delay τ_X, specifically when the dynamic of Y is given as $\frac{dY(t)}{dt} = f(X(t-\tau_X))$, the positive relationship between $X(t-\tau_X)$ and $\dot{Y}(t)$ can be captured by extending the regulation-detection function with a time delay as follows:

$$I_{X+}^{Y}(t,t^*,\tau_X) := X^d(t,t^*,\tau_X) \times \dot{Y}^d(t,t^*) := (X(t-\tau_X) - X(t^*-\tau_X)) \times (\dot{Y}(t) - \dot{Y}(t^*)).$$

In the presence of positive regulation from X to Y with time delay τ_X, $I_{X+}^{Y}(t,t^*,\tau_X)$ is always positive where $X^d(t,t^*,\tau_X) > 0$, and its normalized integral (i.e., extended regulation-detection score with a time delay),

$$S_{X+}^{Y}(\tau_X) := \frac{\iint_{X^d(t,t^*,\tau_X)>0} I_{X+}^{Y}(t,t^*,\tau_X)dtdt^*}{\iint_{X^d(t,t^*,\tau_X)>0} |I_{X+}^{Y}(t,t^*,\tau_X)|dtdt^*} \tag{2}$$

is one.

This idea can be applied to the case of delayed negative regulation. In the presence of negative regulation $X \dashv Y$ with time delay τ_X, the extended regulation-detection function with a time delay,

$$I_{X-}^{Y}(t,t^*,\tau_X) := -X^d(t,t^*,\tau_X) \times \dot{Y}^d(t,t^*) := -(X(t-\tau_X) - X(t^*-\tau_X)) \times (\dot{Y}(t) - \dot{Y}(t^*))$$

can capture the positive relationship between $-X(t-\tau_X)$ and $\dot{Y}(t)$. Thus, $I_{X-}^{Y}(t,t^*,\tau_X)$ is always positive where $-X^d(t,t^*,\tau_X) > 0$, and its normalized integral,

$$S_{X-}^{Y}(\tau_X) := \frac{\iint_{-X^d(t,t^*,\tau_X)>0} I_{X-}^{Y}(t,t^*,\tau_X)dtdt^*}{\iint_{-X^d(t,t^*,\tau_X)>0} |I_{X-}^{Y}(t,t^*,\tau_X)|dtdt^*} \tag{3}$$

is one.

Now, we illustrate how to infer time-delayed causal interactions using a simple example, taken from [16], where X negatively regulates Y with time delay $\tau_X = 3$ (Supplementary Fig. 12a). To infer the delayed regulation, we use the extended regulation-detection score with a time lag (Eq. (2) and (3)). We compute $S_{X-}^{Y}(\tau)$ for each time-series data with different values of τ, varying from 0 to 5 (Supplementary Fig. 12b). $S_{X-}^{Y}(\tau)$ is always one only when the τ is equal to τ_X, whereas if τ is not equal to τ_X, $S_{X-}^{Y}(\tau)$ is not always one. Thus, GOBI can be used to infer delayed regulation and detect the corresponding time delay. This idea can be extended to cases with multi-dimensional regulation, which would be an interesting future direction.

XVI. GUIDELINE FOR DETERMINING WHETHER THE DATA IS SUFFICIENT OR NOT

Here, we suggest a guideline to assist users in determining the amount of data required for GOBI with simple examples. With the ODE describing the regulation (Supplementary Fig. 13a-c):

$$\dot{Y} = \sum_{i=1}^{d} X_i,$$

where d is the dimension of regulation, we simulate 50 time series on $[0,1]$ from different initial conditions. The time series of input signal X_i are constructed by connecting 5 randomly selected points from $[0,1]$ over the time domain with the spline fitting. Among the 50 time-series data, we use the part of them (i.e., M number of data, Supplementary Fig. 13d-f (i)-(v)) to compute the regulation-detection function (I_{X+}^{Y}). Then, we focus on its domain, the regulation-detection region (R_{X+}). As we increase the number of time-series data, the space of the regulation-detection function gradually expands. Once the space fills the entire domain, GOBI can provide a confident result. Achieving full coverage of the domain requires exponentially more data as the dimension of regulation increases, assuming a similar quality of data. For example, when using 10, 25, and 50 numbers of data, the space of I_{X+}^{Y} for 1D, 2D, and 3D regulations are similarly filled, respectively (Supplementary Fig. 13b (iii), c (iv), and d (v)). Thus, in this example, twice as much data is required as the dimension increases by one. In case the available data is not sufficient, i.e., when the domain is not adequately filled, our framework will issue a warning that more data is required for confident result.

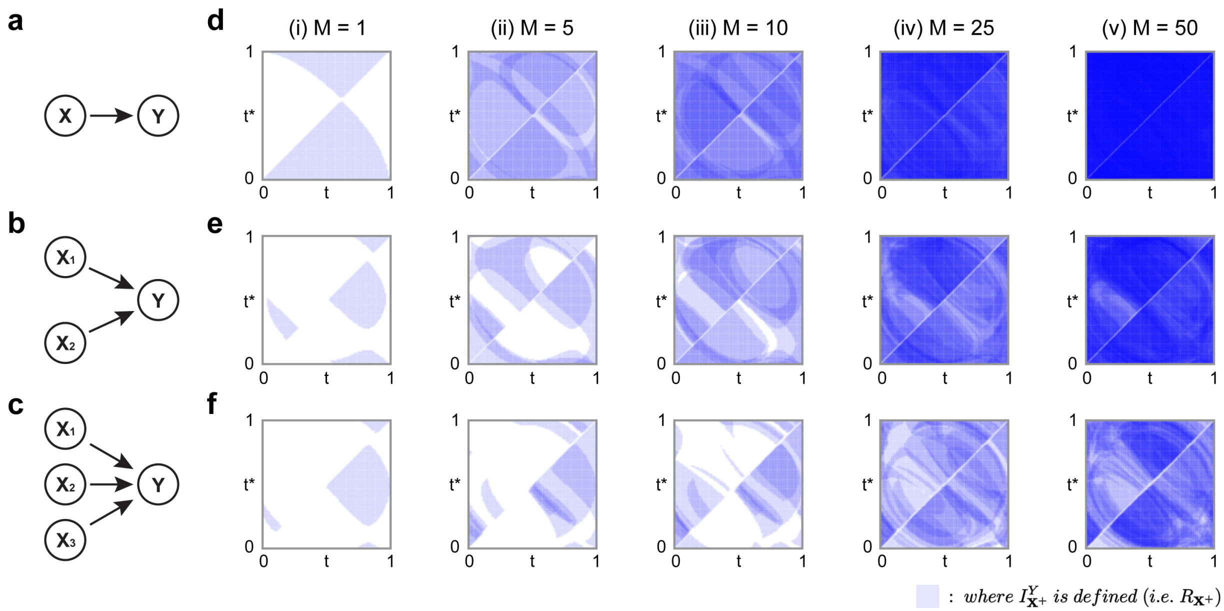

Supplementary Fig. 13. The required amount of data increases as the dimension of regulation increases. **a-c** Three different dimensional regulations from X to Y: 1D regulation (**a**), 2D regulation (**b**), and 3D regulation (**c**). For each regulation, 50 time-series data are simulated on $[0, 1]$ from different initial conditions. **d-f** Using the part of the time-series data (M number of data, **d-f** (i)-(v)), I^Y_{X+} are computed and their domains (i.e., regulation-detection region, R_{X+}) are shown. As the number of data increases, the space of the regulation-detection function gradually expands and fills the entire domain ($[0, 1]^2$) if there is enough data. To fill the entire domain, exponentially more data is required as the dimension increases (**d** (iii), **e** (iv), and **f** (v)). Source data are provided as a Source Data file.

XVII. COMPUTATIONAL COST OF GOBI

In this section, we provide the order of magnitude of runtime for GOBI, assuming N components in the system, M time-series data, and T time points for each time series. To infer every d-dimensional regulation in the system, we perform three steps in GOBI: regulation-detection score, delta test, and surrogate test. First, from a single time-series data, the computation of regulation-detection score has a time complexity of $O(T^2)$. When considering all possible d-dimensional regulations and using M time-series data, the computation of regulation-detection scores has a time complexity of $O(M \times N^d \times T^2)$. Next, in the delta test, for each regulation that satisfies the criteria of the regulation-detection score, we perform the Wilcoxon signed-rank test on every causal variable. Since the Wilcoxon signed-rank test has a time complexity of $O(M^2)$, the delta test has a time complexity of $O(M^2 \times d)$ for a single regulation. Thus, the worst-case time complexity of the delta test is $O(M^2 \times d \times N^d)$. Lastly, in the surrogate test, for each regulation that satisfies the criteria of the delta test, we compute the regulation-detection score with surrogate time series. If the number of surrogate time series is S, then the surrogate test has a time complexity of $O(T^2 \times S \times M)$ for a single regulation. Thus, the worst-case time complexity of the surrogate test is $O(T^2 \times S \times M \times N^d)$. Note that our method can reduce the runtime by utilizing parallel computing.

XVIII. MANUAL FOR THE GOBI COMPUTATIONAL PACKAGE

Here, we provide a manual for a computational package, GOBI (General ODE-Based Inference), to infer a network structure from time-series data. To help user's understanding, we use an illustrated example of the repressilator (Fig. 4c).

1. Generate the 'data.mat' file, which contains two variables 't' and 'y'. 't' is the time points at which the measurements were taken. Each column of 'y' should be the data for each variable at the respective time points (see Input in Supplementary Fig. 14).

2. Run the 'Step0_interpolation_and_cut.m' file. (see Interpolation & Cut in Supplementary Fig. 14). This function

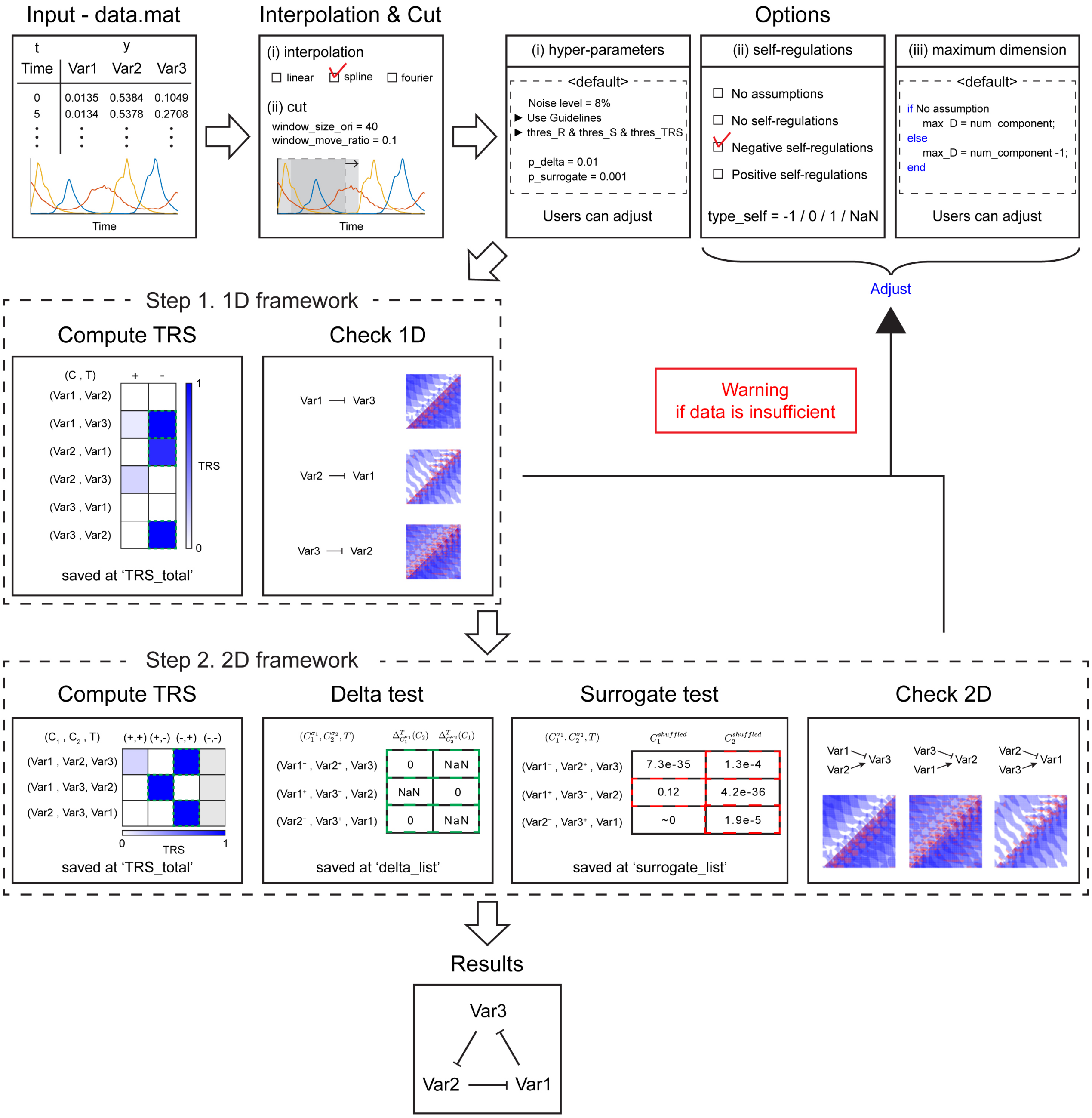

Supplementary Fig. 14. Sample input and output for the GOBI package based on the experimental repressilator example (Fig. 4b). The 'data.mat' file contains the time points ('t') and the time-series data for each variable ('y'). Then, the time series are interpolated using the 'spline' method and cut using the moving window technique. Next, users have options regarding hyper-parameters, types of self-regulation, and the maximum dimension of the framework. Here, we use default values for hyper-parameter, assuming negative self-regulation, and specifying 'max_D = 2'. With these options, our 1D framework computes TRS and infers 1D regulations. Also, our 2D framework computes TRS and performs Δ test and surrogate test to infer 2D regulations. By merging the inferred regulations, a network structure is reconstructed. During the inference, our framework automatically gives a warning signal when the data is insufficient. Source data are provided as a Source Data file.

interpolates the data based on the interpolation method specified by the user and cuts the data into windows using moving window technique, where the window size and overlapping ratio are defined by the user as follows.

(a) Users need to specify the interpolation method using the parameter 'method'. Specifically, 'method = 1' indicates 'linear' interpolation, 'method = 2' indicates 'spline' interpolation, and 'method = 3' indicates 'fourier' interpolation. In the case of 'method = 3', users also have to specify the order of 'fourier' interpolation (i.e., 'num_fourier = 1 to

8'). For less noisy data, 'spline' method is recommended, and for highly noisy data, 'fourier' method with order 2 is recommended.

(b) Users need to choose the sampling rate for the interpolation. The parameter 'time_interval' indicates how finely the users wants to interpolate the original time series. For example, 'time_interval = 0.5' indicates interpolation using a time interval twice as fine as the original time series. Selecting 'time_interval' to make about 100 time points per period is recommended, and please note that the low value of 'time_interval' (high sampling rate) makes the inference accurate, but slow as well.

(c) For the data segmentation, users need to specify the parameters for the moving window technique, i.e., window size and overlapping ratio. The parameter 'window_size_ori' defines the number of time points in each window. Then, along the time series, we move the window until the next window overlaps with the current window by the ratio defined in the parameter 'overlapping_ratio' ('overlapping_ratio = 0.1' as a default). For oscillatory time-series data, it is recommended to choose the window size as one period. The time series in every window is saved at the variable 'y_total'.

After interpolation and data segmentation, the data is saved in 'data_cut.mat' file.

3. Updates the 'Step0_options.m' file (see Options in Supplementary Fig. 14). This code integrates options that can be adjusted by the users or set via our guidelines.

(a) Users need to specify the thresholds for regulation-detection region ('thres_R'), regulation-detection score ('thres_S'), and total regulation score ('thres_TRS') as well as the critical values for Δ test ('p_delta = 0.01' as defaults), and surrogate test ('p_surrogate = 0.001' as defaults). To assist users in selecting those values, we have provided guidelines based on the noise level of data (Supplementary Fig. 5). Thus, users can use our guidelines as defaults or make adjustments depending on whether the goal is to decrease false positive or negative predictions.

(b) The parameter 'type_self' defines options for the types of self-regulation: no assumption ('type_self = NaN'); negative self-regulation ('type_self = -1'); no self-regulation ('type_self = 0'); and positive self-regulation ('type_self = 1'). Also, users can optionally incorporate another available prior knowledge into the inference (Supplementary Fig. 3). Of course, it is possible to run the inference without any prior knowledge, but incorporating such knowledge is beneficial when the amount of data is limited.

(c) The parameter 'max_D' defines the maximum dimension of the framework for inference. Typically, if the system of interest consists of N components, it is recommended to run the inference up to an $N-1$ dimensional framework (N dimensional framework including self-regulations). However, this recommendation is not always feasible because inferring high-dimensional regulation requires a large amount of data. To assists the users in choosing an appropriate dimension of the framework, we have provided the guidelines to check whether the current data is sufficient for running the inference of a specific dimension (Supplementary Fig. 13).

All the options are saved in the 'data_with_options.mat' file. Also, during the inference, our framework automatically gives a warning signal when the data is insufficient to run the framework. Then, users should adjust these options.

4. Run the codes for 1D framework (see Step 1 in Supplementary Fig. 14).

(a) Run the 'Step1_compute_RDS_dim1.m' function. First, this function finds all the possible 1D regulations and saves them at the variable 'component_list_dim1'. Each row of component_list_dim1' indicates the set of causal variable (C) and target variable (T). For each pair (C and T), regulation-detection region and score are computed for all the regulation types (+ and $-$) using time-series data, and they are saved at the variables 'R_total_list' and 'S_total_list'. Those values are saved in the 'RDS_dim1.mat' file.

(b) Run the 'Step1_compute_TRS_dim1.m' function. Using the 'thres_R' and 'thres_S' that users specified, Total Regulation Score (TRS) is computed for each possible 1D regulation. As a result, the heatmap of TRS is displayed, and the exact values of TRS are saved at the variable 'TRS_total' in the 'TRS_dim1.mat' file. In this heatmap, each row indicates the possible 1D regulation (C and T) and each column indicates the regulation type (+ and $-$). Using the 'thres_TRS' that users specified, 1D regulations are inferred.

(c) Run the 'Check1D.m' function. This function checks whether the data is sufficient to confidently infer 1D regu-

lations. If the warning signal comes out, then users are recommended to either stop the inference or adjust the options.

5. Run the codes for 2D framework (see Step 2 in Supplementary Fig. 14).

(a) Run the 'Step2_compute_RDS_dim2.m' function. First, this function finds all the possible 2D regulations and saves them at the variable 'component_list_dim2'. Each row of component_list_dim2' indicates the set of two causal variables (C_1 and C_2), and target variable (T). For each set (C_1, C_2, T), regulation-detection region and score are computed using time-series data for all the regulation types ($(+,+), (+,-), (-,+)$, and $(-,-)$) and they are saved at the variables 'R_total_list' and 'S_total_list'. These values are saved in the 'RDS_dim2.mat' file.

(b) Run the 'Step2_compute_TRS_dim2.m' function. Using the 'thres_R' and 'thres_S' that users specified, Total Regulation Score (TRS) is computed for each possible 2D regulation. As a result, the heatmap of TRS is displayed, and the exact values of TRS are saved at the variable 'TRS_total' in the 'TRS_dim2.mat' file. In this heatmap, each row indicates the possible 2D regulation (C_1, C_2, T) and each column indicates the regulation type ($(+,+), (+,-), (-,+)$, and $(-,-)$). Using the 'thres_TRS' that users specified, candidates for 2D regulations are inferred.

(c) Run the 'Step2_Delta_test_dim2.m' function. For every candidate for 2D regulations (Inferred from 5-(b)), this function performs the Δ test for each causal variable (C_1 and C_2). If the number of data is smaller than 25, then this function tests whether the signs of regulation-delta functions are non-negative or not. If the number of data is larger than 25, this function performs the Wilcoxon signed ranked test. The result of Δ test is saved at the variable 'delta_list' in the 'Delta_dim2.mat' file. Each row of the 'delta_list' represents the candidate for 2D regulation, and two columns of 'delta_list' represents the results of Δ test of for C_1 and C_2. Using the 'p_delta' that users specified, candidates for 2D regulations are inferred.

(d) Run the 'Step2_Surrogate_test_dim2.m' function. For every candidate for 2D regulations (inferred from 5-(c)), this function performs the surrogate test for each causal variable. Users need to specify the number of bootstrapping ('num_boot = 100' as defaults) for the surrogate test. During the simulation, for every time-series data, one of causal variables (C_1 or C_2) is shuffled 'num_boot' times, and regulation-detection scores are computed. Then the p-value is computed for each data and causal variable. Those p-values are combined using Fisher's method. The results of the surrogate test are saved in the variable 'surrogate_list' in the 'Surrogate_dim2.mat' file. Each row of 'surrogate_list' represents the candidate for 2D regulations. The first two columns of 'surrogate_list' represents the results of surrogate test for C_1 and C_2. The next two third and fourth columns of 'surrogate_list' represents the thresholds for combined p-values (combine 'p_surrogate' for all the data). Finally, by using these thresholds, 2D regulations are inferred.

(e) Run the 'Check2D.m' function. This function checks whether the data is sufficient to confidently infer 2D regulations. If the warning signal comes out, then users are recommended to either stop the inference or adjust the options.

These steps are continued until the 'max_D'-dimensional framework. After that, run the function 'Merging_regulations.m' to infer a network structure by merging all the inferred regulations, a network structure is inferred. Since GOBI involves multi-dimensional inferences, it is possible to detect various dimensional regulations for a single target. In this case, GOBI infers the regulation with the highest value of TRS. Here, we illustrate up to the 2D framework, but users can easily expand this approach to include higher dimensions as needed (see Github codes (https://github.com/Mathbiomed/GOBI) [17] for Fig. 4).

SUPPLEMENTARY REFERENCES

[1] Kim, J. K. & Forger, D. B. A mechanism for robust circadian timekeeping via stoichiometric balance. *Molecular systems biology* **8**, 630 (2012).

[2] Igoshin, O. A., Goldbeter, A., Kaiser, D. & Oster, G. A biochemical oscillator explains several aspects of myxococcus xanthus behavior during development. *Proceedings of the National Academy of Sciences* **101**, 15760–15765 (2004).

[3] Goodwin, B. C. Oscillatory behavior in enzymatic control processes. *Advances in enzyme regulation* **3**, 425–437 (1965).

[4] Goldbeter, A. A model for circadian oscillations in the drosophila period protein (per). *Proceedings of the Royal Society of London. Series B: Biological Sciences* **261**, 319–324 (1995).

[5] Maeda, M. *et al.* Periodic signaling controlled by an oscillatory circuit that includes protein kinases erk2 and pka. *Science* **304**, 875–878 (2004).

[6] Mangan, S. & Alon, U. Structure and function of the feed-forward loop network motif. *Proceedings of the National Academy of Sciences* **100**, 11980–11985 (2003).

[7] Gotoh, T. *et al.* Model-driven experimental approach reveals the complex regulatory distribution of p53 by the circadian factor period 2. *Proceedings of the National Academy of Sciences* **113**, 13516–13521 (2016).

[8] Lillacci, G. & Khammash, M. Parameter estimation and model selection in computational biology. *PLoS computational biology* **6**, e1000696 (2010).

[9] Toni, T., Welch, D., Strelkowa, N., Ipsen, A. & Stumpf, M. P. Approximate bayesian computation scheme for parameter inference and model selection in dynamical systems. *Journal of the Royal Society Interface* **6**, 187–202 (2009).

[10] Veilleux, B. G. The analysis of a predatory interaction between didinium and paramecium (1976).

[11] Aufinger, L., Brenner, J. & Simmel, F. C. Complex dynamics in a synchronized cell-free genetic clock. *Nature communications* **13**, 1–9 (2022).

[12] Potvin-Trottier, L., Lord, N. D., Vinnicombe, G. & Paulsson, J. Synchronous long-term oscillations in a synthetic gene circuit. *Nature* **538**, 514–517 (2016).

[13] Métivier, R. *et al.* Estrogen receptor-α directs ordered, cyclical, and combinatorial recruitment of cofactors on a natural target promoter. *Cell* **115**, 751–763 (2003).

[14] Wong, T. W. *et al.* Air pollution and hospital admissions for respiratory and cardiovascular diseases in hong kong. *Occupational and environmental medicine* **56**, 679–683 (1999).

[15] Leng, S. *et al.* Partial cross mapping eliminates indirect causal influences. *Nature communications* **11**, 1–9 (2020).

[16] Glass, D. S., Jin, X. & Riedel-Kruse, I. H. Nonlinear delay differential equations and their application to modeling biological network motifs. *Nature communications* **12**, 1788 (2021).

[17] Park, S. H., Ha, S. & Kim, J. K. A general model-based causal inference method overcomes the curse of synchrony and indirect effect. Mathbiomed/GOBI: GOBI (General ODE-based causal inference) (v1.0.0). *Zendo* (2023). URL https://doi.org/10.5281/zenodo.7997213.
