# Supplementary material for: A general model-based causal inference method overcomes the curse of synchrony and indirect effect
Source: Nat Commun. 2023 Jul 24;14:4287. doi: 10.1038/s41467-023-39983-4 (PMC10366229; doi:10.1038/s41467-023-39983-4)
Supplement: Supplementary file 3 — Description of Additional Supplementary Files [file 41467_2023_39983_MOESM3_ESM.pdf]

Description of Additional Supplementary Files:

Supplementary Data 1: Inference results from various *in silico* systems.
